# Single‐Cell Transcriptomic Analysis of Tumor Heterogeneity and the Microenvironment in Pseudomyxoma Peritonei

**DOI:** 10.1002/advs.202523760

**Published:** 2026-07-27

**Authors:** Xi Li, Lei Wang, Lei Ai, Zimeng Li, Zhaochangci Chen, Yuchen Luo, Tianran Zhang, Mengjia Liao, Songhao Wang, Jiajia Cui, Ke Liu, Chengxian Guo, Guodong Liu, Jiye Yin, Wei Wu

**Affiliations:** ^1^ Department of Geriatric Surgery Xiangya Hospital Central South University Changsha Hunan People's Republic of China; ^2^ National Clinical Research Center for Geriatric Disorders Changsha Hunan People's Republic of China; ^3^ Department of General Surgery Xiangya Hospital Central South University Changsha Hunan People's Republic of China; ^4^ FuRong Laboratory Changsha Hunan People's Republic of China; ^5^ Liangzhu Laboratory Zhejiang University Hangzhou People's Republic of China; ^6^ Department of Ophthalmology The Third Xiangya Hospital Central South University Changsha Hunan People's Republic of China; ^7^ Department of Clinical Pharmacology Xiangya Hospital Central South University Changsha Hunan People's Republic of China; ^8^ Institute of Clinical Pharmacology Hunan Key Laboratory of Pharmacogenetics Central South University Changsha Hunan People's Republic of China; ^9^ Engineering Research Center of Applied Technology of Pharmacogenomics Ministry of Education Changsha Hunan People's Republic of China; ^10^ Center of Clinical Pharmacology the Third Xiangya Hospital Central South University Changsha Hunan People's Republic of China; ^11^ Xiangya School of Medicine Central South University Changsha Hunan People's Republic of China

**Keywords:** anti‐angiogenic therapy, appendiceal mucinous neoplasm, immune exclusion, lipid metabolism, pseudomyxoma peritonei, single‐cell RNA sequencing, tumor microenvironment

## Abstract

Pseudomyxoma peritonei (PMP) is characterized by progressive mucus accumulation, extensive stromal fibrosis, rare extraperitoneal metastasis, limited therapeutic options, and frequent recurrence. However, the microenvironmental ecosystem of PMP, particularly in metastatic lesions, remains poorly understood. Here, we integrated single‐cell RNA sequencing, whole‐exome sequencing, bulk RNA sequencing, and histopathologic validation to construct a high‐resolution atlas of primary and paired metastatic tumors. Epithelial cells showed distinct functional states, including a TFF3^+^ mucus secretion‐associated state and a MACC1^+^ malignant‐associated state. Metastatic lesions showed coordinated microenvironmental reprogramming, including POSTN^+^ fibrosis‐associated fibroblasts, CXCL5^+^ macrophages linked to local immunosuppressive signaling, and immune exclusion associated with a collagen‐rich stromal barrier. We also observed extensive lipid metabolic activity and identified a candidate pro‐angiogenic network involving POSTN^+^ fibroblasts, RSPO3^+^ pericytes, and endothelial cells, potentially mediated by VEGFA–VEGFR2 signaling. Retrospective observations from three recurrent PMP cases further suggested the potential therapeutic value of VEGFR2‐targeted anti‐angiogenic therapy. Overall, this study provides a comprehensive single‐cell transcriptomic atlas of PMP and a resource for developing novel and combination therapeutic strategies.

## Graphical abstract

This study presents a single‐cell atlas of pseudomyxoma peritonei spanning primary and paired metastatic lesions. Distinct epithelial substates, stromal remodeling, immune exclusion, lipid metabolic reprogramming, and a candidate angiogenic network were identified in metastatic lesions. These findings advance the understanding of PMP biology and provide a resource for the development of novel targeted and combination treatment strategies.

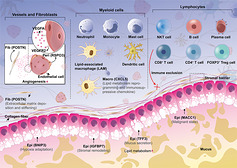

## 1 Introduction

Pseudomyxoma peritonei (PMP) is characterized by progressive accumulation and redistribution of mucinous tumor deposits within the peritoneal cavity [1, 2]. Although reported worldwide, population‐based studies suggest geographic variation in disease burden, with lower incidence in East Asia and higher rates in Europe [3, 4]. Advances in disease awareness, standardized pathological classification and diagnostic terminology, and specialized treatment centers have collectively reduced underdiagnosis and improved detection [5]. Most cases originate from appendiceal mucinous neoplasms (AMN), and a minority from mucinous tumors of the ovary, colon, pancreas, or other organs [6, 7, 8]. After progressive intraluminal mucus accumulation and rupture of the primary tumor (PT), tumor cells disseminate with mucus throughout the peritoneal cavity and implant widely, giving rise to metastatic tumors (MT). Because progression is often insidious, patients are frequently diagnosed at an advanced stage, and symptoms from mucus accumulation and fibrosis‐mediated organ compression are a major cause of clinical presentation and therapeutic challenge [9].

Cytoreductive surgery (CRS) with hyperthermic intraperitoneal chemotherapy (HIPEC) is the standard first‐line treatment for PMP [10]. Because PMP typically remains confined to the peritoneal cavity, with limited invasion of deep organs and rare extraperitoneal metastasis, appropriately treated patients may achieve favorable long‐term survival, with reported 5‐year overall survival up to 57.8% [11]. However, CRS is highly invasive and technically demanding, often requiring multivisceral resection and prolonged operative time, yet it rarely achieves complete tumor eradication. Postoperative recurrence remains common, and many patients require repeated surgery, imposing substantial physical, psychological, and financial burden, thereby adversely affecting quality of life. Conventional systemic chemotherapy has shown limited efficacy [12], and no targeted or immunotherapeutic agents are approved for PMP. For patients with advanced disease or poor surgical tolerance, effective options remain scarce and outcomes unsatisfactory. These limitations underscore the urgent need to elucidate the molecular basis of core PMP phenotypes and identify novel therapeutic opportunities.

Previous omics studies have laid the foundation for understanding the molecular landscape of PMP. Genomic studies identified KRAS and GNAS as the most frequent somatic driver mutations, with less frequent alterations in TP53, SMAD4, BRAF V600E, and APC [13, 14, 15, 16]. Functional studies targeting KRAS G12D or BRAF V600E inhibited PMP progression in disease models [17, 18]. Nevertheless, the mechanisms by which these alterations drive key phenotypes such as excessive mucus production remain poorly defined, and their prognostic relevance is debated [16]. At the transcriptomic level, bulk‐tissue studies showed enrichment of angiogenesis, epithelial‐mesenchymal transition (EMT), and inflammatory response pathways in PMP tumors [19]. However, mixed tissue‐level signals cannot resolve the heterogeneity, type‐specific contributions, and interactions of tumor and non‐tumor compartments within the microenvironment, which is particularly important in PMP, where tumor cellularity is low and stromal and mucinous components are abundant.

PMP shares several biological features with other mucinous or peritoneal dissemination‐associated malignancies, including excessive mucus production, extensive stromal remodeling, and adaptation to the peritoneal microenvironment. The use of CRS with HIPEC across multiple peritoneal malignancies further suggests partially convergent pathological and therapeutic principles [20, 21]. Thus, systematic dissection of PMP biology may not only improve disease‐specific management but also provide broader insight into the mechanisms and treatment vulnerabilities of mucinous tumors and peritoneal metastatic diseases.

Single‐cell RNA sequencing (scRNA‐seq), which resolves transcriptional states at the single‐cell level, has uncovered key cellular subpopulations, developmental trajectories, and functional programs across diverse tumor types [22, 23, 24, 25]. Recently, two studies extended this approach to PMP. Ayala et al. [26] profiled AMN and PMP metastases at single‐cell resolution, defined epithelial gene signatures, and highlighted a goblet cell‐like tumor epithelial identity together with programs related to RAS signaling, EMT, and altered lipid metabolism. Ha et al. [27] generated an integrated resource combining scRNA‐seq and bulk RNA sequencing (bulk RNA‐seq) from PMP metastases, emphasizing the coexistence of epithelial and mesenchymal features. However, neither study focused on paired primary and metastatic lesions from the same patient, which are important for directly comparing programs of peritoneal dissemination and metastatic adaptation. Thus, high‐resolution analysis of paired lesions is still needed to define the metastatic ecosystem of PMP.

Here, we aimed to comprehensively characterize the cellular and molecular ecosystems of PT and paired MT in PMP using integrated single‐cell and bulk multi‐omics approaches. This work delineated functionally distinct epithelial states, highlighted lipid metabolism‐associated programs, characterized stromal and immune features of the microenvironment, and identified candidate angiogenic interaction networks. Together, this study provides a refined, complementary framework for understanding PMP progression and a paired‐lesion single‐cell resource that may inform the development of new therapeutic strategies.

## 2 Materials and Methods

### 2.1 Sample Collection and Tissue Dissociation

All human tissue collection protocols were approved by the Ethics Committee of Xiangya Hospital, Central South University (approval no. 2024101296), and written informed consent was obtained from each participant. All PMP samples were surgically resected at our institution and pathologically confirmed before inclusion.

Fresh surgical specimens were dissected to isolate representative tumor regions, then immediately placed in pre‐chilled tissue preservation solution (130‐100‐008, Miltenyi) and transported to the laboratory. In a biosafety cabinet, tissues were cut into 2–3 mm^3^ fragments and gently rinsed with PBS. Fragments were transferred into tubes containing tissue dissociation buffer (130‐095‐929, Miltenyi Biotec) and incubated at 37°C with agitation until no obvious clumps remained (≤45 min). The digested suspension was filtered through a 70 µm MACS smart strainer (130‐098‐462, Miltenyi Biotec), followed by centrifugation at 400 × g for 10 min at 4°C. After supernatant removal, red blood cell lysis buffer (R1010‐500, Solarbio) was added on ice for 3–5 min, then quenched with PBS, and centrifuged again at 400 × g for 10 min at 4°C. The pellet was resuspended at a final concentration of 800–1000 cells/µL. Only suspensions with cell viability >90% and aggregation rate <15% were used for library preparation.

### 2.2 10x ScRNA‐seq

Following the manufacturer's instructions for the Chromium Next GEM Single Cell 3' Reagent Kits (v3.1), the prepared single‐cell suspension was loaded onto the Chromium Single Cell Controller (10x Genomics), targeting ∼10,000 captured cells per sample. After library construction, quantification was performed using a Qubit 4.0 fluorometer. Libraries were diluted to 1 ng/µL and assessed for insert size on an Agilent 2100 Bioanalyzer. Qualified libraries were quantified on a Bio‐Rad CFX 96 real‐time PCR system, requiring an effective concentration above 10 nM. Sequencing used the Illumina NovaSeq 6000 platform using paired‐end 150‐bp reads.

### 2.3 Quality Control and Processing of scRNA‐seq Data

Raw sequencing data were aligned to the GRCh38 reference genome and quantified with Cell Ranger (v3.1.0, 10x Genomics). Filtered count matrices were imported into Seurat (v5.2.1) [28]. Quality control excluded cells with fewer than 100 detected genes, fewer than 500 total UMI counts, or mitochondrial proportions above 15%. Doublets were removed using DoubletFinder (v2.0.3) [29].

Filtered data were processed with the SCTransform workflow for normalization and variance stabilization; no Seurat anchor‐based integration was performed. Principal component analysis (PCA) was performed on the SCT assay, followed by Harmony correction of PCA embeddings to mitigate sample‐ or batch‐associated effects. Clustering used a shared nearest‐neighbor graph and the FindClusters function. To select clustering granularity, we compared multiple resolutions and evaluated the resulting structures by cluster‐tree continuity, marker‐gene coherence, cluster size, and biological interpretability. A working clustering resolution was then selected on the basis of this multi‐resolution assessment. RunUMAP function performed dimensionality reduction and visualization (Uniform Manifold Approximation and Projection, UMAP; dims = 20, n.neighbors = 30, min.dist = 0.3).

### 2.4 Cell‐Type Annotation

Major cell types were initially annotated using canonical marker genes. To support annotation, differentially expressed genes for each cell type were identified with FindAllMarkers in Seurat using Bonferroni‐corrected Wilcoxon rank‐sum tests, considering genes with adjusted *p* < 0.05 as significant. Additional thresholds required expression in at least 25% of cells in the target cluster (min.pct > 0.25) and a log fold‐change above 0.25 versus all other cells (logfc.threshold > 0.25). Final annotation of each subcluster integrated canonical markers with cluster‐specific differential expression profiles.

### 2.5 Copy Number Variation (CNV) Inference in scRNA‐seq

Large‐scale CNV patterns in epithelial cells were inferred with inferCNV (v1.22.0) [30], using immune cells as reference. Single‐cell CNV signals were estimated with a default‐length sliding window, and CNV burden was calculated per epithelial cell. To stratify cells by CNV burden, the pooled score distribution was fitted with a three‐component Gaussian mixture model. Two global cutoffs, defined as midpoints between adjacent component means, classified cells as CNV‐normal, CNV‐low, or CNV‐high. These cutoffs were applied uniformly across all samples without per‐sample refitting.

### 2.6 Pathway Enrichment and Metabolic Pathway Analysis

Gene Ontology (GO) and Kyoto Encyclopedia of Genes and Genomes (KEGG) enrichment analyses used clusterProfiler (v4.12.6) [31], with terms at adjusted *p* < 0.05 considered significantly enriched. Single‐cell metabolic activity was analyzed with scMetabolism (v0.2.1) [32]: using the normalized expression matrix from Seurat objects and Reactome metabolic pathways as reference sets, activity scores were calculated with the AUCell algorithm.

### 2.7 Pseudotime Analysis

Pseudotime trajectory analysis used Monocle2 (v2.24.0) [33]. The newCellDataSet function constructed the CellDataSet object using a negative binomial model (negbinomial.size) with a lower detection limit of 0.5. Differential analysis used differentialGeneTest (reducedModelFormulaStr = “∼1”) to screen differentially expressed genes (FDR < 0.001). Dimensionality reduction was used with the reduceDimension function, and trajectories were inferred with the DDRTree algorithm (norm_method = none, pseudo_expr = 0, param.gamma = 10) and visualized with the plot_cell_trajectory function.

### 2.8 Identification of Gene Expression Programs (GEPs)

GEPs reflecting cell‐type characteristics and functional activities were identified with the standard consensus non‐negative matrix factorization (cNMF) (v1.7.0) workflow [34]. The prepare module preprocessed the single‐cell expression matrix, and the factorize module ran non‐negative matrix factorization across parallel tasks to obtain repeated results under different factor numbers. A 2D k_selection_plot diagnostic was used to select the optimal factor number. Finally, the consensus module (local‐density‐threshold = 0.1) performed consensus clustering and component extraction for the selected factor number, yielding stable GEPs.

### 2.9 SCENIC Regulon Specificity Analysis

Transcription factor regulatory network analysis used the pySCENIC workflow (v0.12.1) [35]. From the gene expression matrix (SCT‐normalized counts), the pyscenic grn module identified candidate transcription factor‐target networks by co‐expression, and the pyscenic ctx module performed motif enrichment. Motif‐ranking databases and motif‐to‐transcription factor annotations were obtained from the cisTarget resources website (https://resources.aertslab.org/cistarget/), generating candidate regulons. Regulon activity per cell was quantified with the pyscenic aucell module (AUCell algorithm), applying auc_threshold = 0.1 to reduce background noise. Regulon specificity scores were then calculated with the calcRSS function across cell subclusters.

### 2.10 Intercellular Communication Analysis

Cell‐cell communication was analyzed with CellChat (v1.6.1) using the human CellChatDB database [36]. Remarkably, overexpressed signaling molecules and ligand‐receptor interactions were identified with the identifyOverExpressedGenes and identifyOverExpressedInteractions functions, respectively. The computeCommunProb function calculated communication probabilities between cell types, filtering interactions with low cell counts (min.cells = 10). Significant networks were aggregated and visualized using the standard CellChat workflow.

### 2.11 Whole‐Exome Sequencing (WES)

Given the low tumor purity of PMP lesions, ultra‐deep WES at a mean depth of 2000 × was performed to improve sensitivity for low‐frequency somatic variants [37]. Exonic regions were captured with the AIExome Human Exome Panel V3 kit (iGeneTech). Raw data were processed with fastp (v0.21.0) to remove adapter‐contaminated and low‐quality reads. Clean reads were aligned to the UCSC hg38 reference genome with BWA‐MEM (v0.7.17), followed by sorting and duplicate removal with Sambamba (v1.01) at default parameters. Indels were identified with Manta (v1.6.0), and somatic mutations with Strelka (v2.9.10). Variants were annotated with ANNOVAR, visualized with maftools (v2.20.0), and retained if supported by at least five deduplicated reads. Somatic copy number alterations were detected with CNVkit (v0.9.12).

### 2.12 Bulk RNA Sequencing

Total RNA was isolated from tumor tissues with the FastPure Complex Tissue/Cell Total RNA Isolation Kit (Vazyme). Bulk RNA‐seq libraries were prepared with the Hieff NGS Ultima Dual‐mode mRNA Library Prep Kit (Yeasen). Raw reads were filtered with fastp (v0.21.0) and aligned to the UCSC hg38 reference genome with HISAT2 (v2.2.1). StringTie (v3.0.1) quantified transcripts to generate raw counts, FPKM, and TPM. To estimate cellular composition, deconvolution used CIBERSORTx (v0.1.0) [38], with the top 30 feature genes per scRNA‐seq‐defined subcluster as reference signatures and 1,000 permutations for significance.

### 2.13 Hematoxylin‐Eosin (HE) and Alcian Blue (AB) Staining

Tissues were fixed in 4% paraformaldehyde, paraffin‐embedded, and sectioned at 5 µm. For histopathology, sections were deparaffinized, rehydrated, and stained with HE using a commercial kit (G1120, Solarbio) per the manufacturer's instructions. Acidic mucopolysaccharide was visualized with an Alcian blue kit (G1560, Solarbio). Sections were then dehydrated, cleared in xylene, and mounted for microscopy.

### 2.14 TSA‐Based Multiplex Immunohistochemistry (TSA‐mIHC) Staining

TSA‐mIHC staining was performed on formalin‐fixed, paraffin‐embedded tissue sections (5 µm). After deparaffinization and rehydration, sections underwent antigen retrieval in EDTA alkaline solution (pH 8.0) by microwave heating. After cooling, sections were washed with PBST (pH 7.4) for 3 × 5 min with shaking. Endogenous peroxidase activity was blocked with 3% H_2_O_2_ for 15 min at room temperature, followed by 3 × 5 min washes with PBST. Sections were blocked with 100 µL of goat serum per slide (sufficient to cover the tissue) for 30 min at room temperature. Primary antibodies were incubated overnight at 4°C, followed by 3 × 5 min washes with PBST, and then incubated with HRP‐polymer‐conjugated secondary antibody for 30 min at room temperature, followed by 3 × 5 min washes with PBST. TSA fluorophore (e.g., TYR‐480, 520, 570, 620) was applied for 5 min at room temperature, then washed 3 × 5 min with PBST. Between rounds, the entire cycle (antigen retrieval to TSA deposition) was repeated for each additional marker. After the final round, sections were incubated with DAPI in the dark for 10 min, washed 3 × 5 min with PBST, and mounted with antifade medium for fluorescence imaging. Primary antibodies used for multiplex staining included MACC1 (SAB3500273, Sigma), PanCK (AF20164, AiFang), FABP1 (68227‐1‐Ig, Proteintech), TFF3 (23277‐1‐AP, Proteintech), MUC2 (AFRM0229, AiFang), CD4 (AFRM0003, AiFang), CD8 (AF20211, AiFang), Collagen I (AFRP0017, AiFang), FOXP3 (AFRM0039, AiFang), CD68 (AFRM0021, AiFang), CXCL5 (10809‐1‐AP, Proteintech), CD31 (AFRM0001, AiFang), VEGFA (AFRM0295, AiFang), VEGFR2 (AF20357, AiFang), RGS5 (11590‐1‐AP, Proteintech), RSPO3 (66314‐1‐Ig, Proteintech), α‐SMA (AF20323, AiFang), Periostin (AFRM0263, AiFang), and APOE (AFRM0343, AiFang).

### 2.15 Immunohistochemistry (IHC) Staining

For IHC staining, formalin‐fixed, paraffin‐embedded tissue sections (5 µm) were deparaffinized, rehydrated, and subjected to antigen retrieval according to standard procedures. After blocking endogenous peroxidase activity and nonspecific binding, sections were incubated overnight at 4°C, followed by appropriate HRP‐conjugated secondary antibodies and DAB development. Slides were counterstained with hematoxylin, dehydrated, and mounted for bright‐field imaging.

For the quantitative analyses of CD31, VEGFA, and VEGFR2, three independent patient samples were analyzed for each of the non‐neoplastic appendix (NNA), PT, and MT groups. The NNA samples used for IHC included the single NNA specimen incorporated into the scRNA‐seq dataset, together with two additional NNA specimens collected independently.

### 2.16 Statistical Analysis

Statistical analyses used R (v4.4.3; R Foundation for Statistical Computing, Vienna, Austria) and GraphPad Prism (v10.1.2; GraphPad Software, Boston, MA, USA). Continuous variables are presented as mean ± standard error of the mean (SEM) for approximately normally distributed data or median with interquartile range (IQR) for non‐normally distributed data. Data distribution and homogeneity of variance were assessed before testing.

For scRNA‐seq between‐group comparisons, biological samples rather than individual cells were the unit of replication to avoid pseudoreplication: per‐sample summary values were calculated and then compared across tissue groups. Because the scRNA‐seq cohort included only one NNA reference sample, the NNA group was excluded from formal between‐group testing and shown for descriptive reference only.

For two groups, a two‐tailed Student's *t*‐test was used when normality and homogeneity of variance held, Welch's *t*‐test when the equal‐variance assumption was violated, and the Wilcoxon rank‐sum test for non‐normally distributed data. For multiple groups, one‐way ANOVA was used when normality and homogeneity of variance held, followed by Tukey's multiple‐comparisons test; Welch's one‐way ANOVA was applied when equal variances were violated. For nonparametric data, the Kruskal‐Wallis test with Dunn's multiple‐comparisons test was used. All tests were two‐sided, with *p* < 0.05 considered significant. The statistical test, sample size, data presentation, and significance annotations are provided in the corresponding figure legends.

## 3 Results

### 3.1 High‐Resolution Single‐Cell Atlas and Cell Types of Primary Appendiceal Mucinous Tumors and Abdominopelvic Metastatic Tumors

**FIGURE 1:**
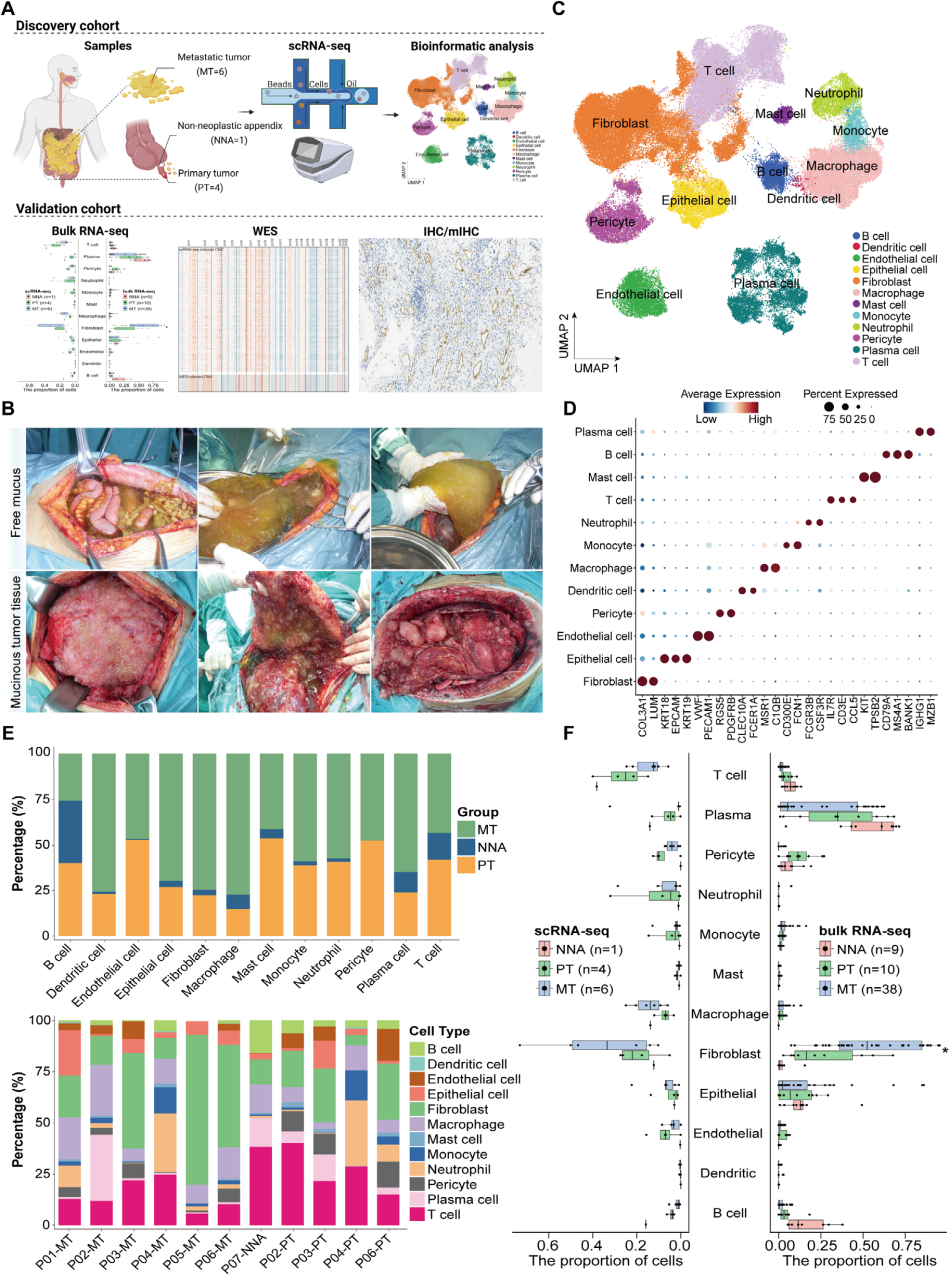
Single‐cell atlas of primary appendiceal tumors and abdominopelvic metastatic tumors in PMP. (A) Schematic overview of the overall study design. Several representative panels incorporated into this schematic are reused from Figure 1C, Figure 1F, Figure S3A, and Figure 7E for illustrative purposes only; these reused panels are not presented as independent experimental results and do not carry separate quantification or interpretation in this panel. (B) Representative intraoperative gross images of abdominopelvic manifestations showing the coexistence of abundant free mucus and mucinous tumor tissue. (C) UMAP plot of the integrated single‐cell atlas from 11 samples (NNA = 1, PT = 4, MT = 6), identifying 12 major cell types. (D) Dot plot showing representative marker genes across major cell types. Color intensity indicates relative expression levels, and dot size indicates the proportion of cells expressing each gene. (E) Stacked bar plots show the relative proportion of tissue origins across major cell types (top), and that of major cell types across individual samples (bottom). (F) Box plots comparing the relative abundance of major cell populations across tissue origins in the scRNA‐seq cohort (left; NNA = 1, PT = 4, MT = 6) and the bulk RNA‐seq validation cohort inferred by CIBERSORTx deconvolution (right; NNA = 9, PT = 10, MT = 38). Data are presented as median with IQR. Statistical analysis of the comparison between the PT and MT groups was performed using the Wilcoxon rank‐sum test. ^*^ *p* < 0.05. The box range represents the IQR, the central horizontal line represents the median, and the whiskers (structures outside the box) extend to 1.5 × IQR.

To analyze PMP at single‐cell resolution, 10 samples were collected from fresh surgically resected tumor tissues of 6 PMP patients (P01‐P06) for scRNA‐seq (10x Genomics). These included PT and matched MT specimens from 4 patients (P02, P03, P04, and P06) and isolated MT from 2 additional patients (P01 and P05). One NNA reference sample was collected from a patient (P07) who underwent right hemicolectomy for a hepatic flexure colon tumor; as it was not from a healthy donor and was the only such specimen, it was included as a descriptive reference rather than a formal normal control. In total, scRNA‐seq data were generated from 11 samples of 7 patients (1 NNA, 4 PT, and 6 MT; Figure 1A; Figure S1A and Table S1). In parallel, WES was performed on MT and matched peripheral blood from five PMP patients (P01, P02, P03, P05, and P06); P04 was excluded as no residual sample remained. Owing to limited lesion size, no PT sample yielded sufficient material for assays other than scRNA‐seq.

The abdominopelvic manifestations of PMP consisted of mucinous tumor tissue with abundant free mucus secreted by it (Figure 1B); here, MT refers specifically to mucinous tumor tissue, not free acellular mucus. Histologically, this tissue comprises cystic structures of variable size with intercystic stromal components: a sparse layer of tumor epithelial cells lines the inner cyst surface, whereas the lumen contains intracystic mucus secreted by these cells (Figure S1B). For scRNA‐seq, intact blocks of mucinous tumor tissue were collected and processed directly, without microdissection or compartment enrichment, and therefore contained intracystic mucus, tumor epithelial cells, cyst walls, and stromal components.

Following quality filtering, 102,717 cells were retained. Multi‐resolution comparison indicated that lower resolutions merged distinct populations, whereas higher resolutions fragmented coherent groups. Resolution 2.0 was selected because it preserved stable parent lineage structure while resolving meaningful substructure (Figure S1C). At this resolution, unsupervised clustering with marker‐gene annotation identified 12 major cell types: epithelial cells, fibroblasts, T cells, plasma cells, B cells, monocytes, macrophages, neutrophils, mast cells, dendritic cells, endothelial cells, and pericytes (Figure 1C,D; Figure S1D). Among these cell types, fibroblasts and T cells were relatively enriched. Notably, fibroblasts showed a trend toward increased abundance in MT compared with PT, whereas T cells exhibited the opposite pattern (Figure 1E,F). Epithelial cells accounted for a small fraction across all samples, averaging less than 10%, consistent with sparse epithelial distribution on histopathology (Figure S1B). These patterns varied substantially across samples and should be interpreted cautiously, given sampling, dissociation, and capture bias. To validate these findings, we included an independent cohort of 18 patients (P08‐P25) operated at our center, comprising 14 with PMP (P08‐P21) and 4 non‐PMP patients (P22‐P25) (Figure S1E and Table S2). Bulk RNA‐seq from this cohort (*n* = 57; 9 NNA, 10 PT, and 38 MT) with CIBERSORTx deconvolution showed relative abundance patterns of major populations largely consistent with the scRNA‐seq analysis (Figure 1F), supporting the overall ecosystem structure.

Collectively, these analyses establish a high‐resolution single‐cell atlas of PMP and describe a heterogeneous tumor ecosystem.

### 3.2 Functional Characterization and Lipid Metabolism Preference of Epithelial Subclusters

**FIGURE 2:**
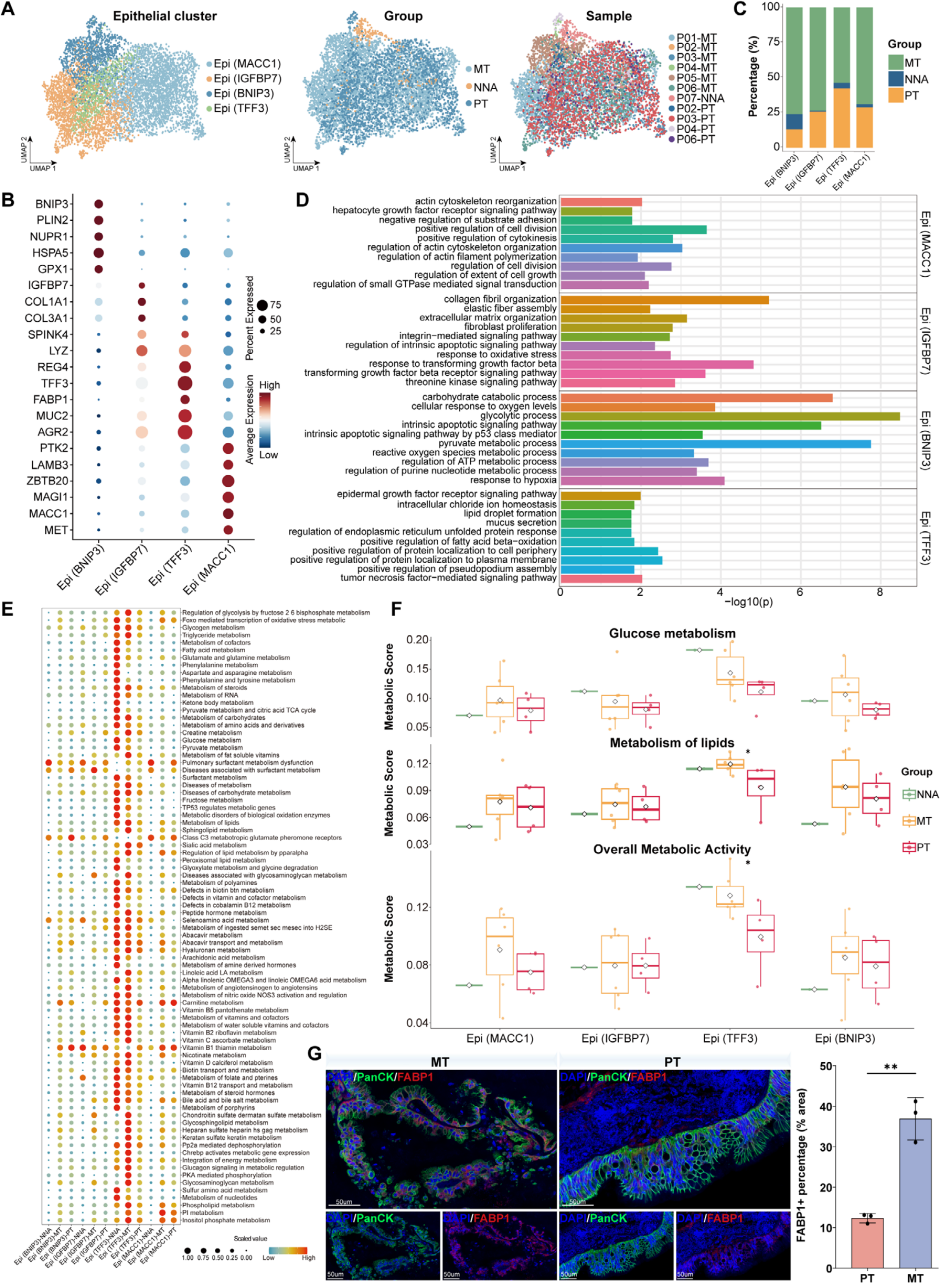
Transcriptional characteristics and active lipid metabolism of epithelial cells. (A) UMAP plot showing the distribution of epithelial cell subclusters (left), tissue origins (middle), and samples (right). (B) Dot plot showing representative marker genes for each epithelial subcluster. (C) A stacked bar plot shows the relative proportion of tissue origins across epithelial subclusters. (D) GO enrichment analysis showing the top 10 significantly enriched biological processes for each epithelial subcluster. (E) Dot plot displays metabolic pathway activity of the four epithelial subclusters across tissue origins based on scMetabolism analysis. Color intensity indicates relative metabolic activity, and dot size represents the proportion of metabolically active cells. (F) Box plots display glucose metabolism activity (top), lipid metabolism activity (middle), and overall metabolic activity (bottom) of epithelial subclusters across tissue origins. Data are presented as median with IQR. Statistical analysis of the comparison between the PT (*n* = 4) and MT (*n* = 6) groups was performed using the Wilcoxon rank‐sum test. ^*^ *p* < 0.05. The box range represents the IQR, the central horizontal line represents the median, and the whiskers extend to 1.5 × IQR. (G) mIHC shows colocalization of DAPI (blue), PanCK (green), and FABP1 (red) in PT and MT samples. Scale bars, 50 µm. The accompanying bar plot shows the quantification results of three independent patient samples per group. Three representative fields were quantified per sample, and the averaged value for each sample was used for statistical analysis. Data are presented as mean ± SEM. Statistical analysis was performed using a two‐tailed Student's *t*‐test. ^**^ *p* < 0.01.

Given the central role of epithelial cells in PMP development and progression, we systematically analyzed the tumor epithelial population. Integration of scRNA‐seq data from all 11 samples identified 6,568 epithelial cells, divided into four subclusters: Epi (BNIP3), Epi (IGFBP7), Epi (TFF3), and Epi (MACC1) (Figure 2A), each with a distinct signature gene expression profile (Figure 2B; Figure S2A). By distribution, Epi (BNIP3) was the major subcluster in the NNA sample, whereas the other three predominated in PT and MT (Figure 2A,C).

To define the biological functions of these subclusters, we performed pathway enrichment analysis (Figure 2D and Figure S2B). Epi (BNIP3) was preferentially associated with hypoxia‐adaptation pathways, such as “Glycolysis/Gluconeogenesis,” “HIF‐1 signaling pathway,” and “response to hypoxia.” Epi (IGFBP7) was associated with “ECM‐receptor interaction” and “transforming growth factor‐beta receptor signaling pathway,” suggesting involvement in stromal remodeling. MUC2, the predominant mucin in PMP, maintains the gel‐like state of the mucus [2]; IHC and AB staining showed strong cytoplasmic MUC2 in tumor epithelial cells, secreted extracellularly to form mucin pools in MT (Figure S2C). Epi (TFF3) showed high MUC2 expression and signatures of mucus secretion and lipid metabolism, including “tumor necrosis factor‐mediated signaling pathway,” “epidermal growth factor receptor signaling pathway,” “p53 signaling pathway,” “positive regulation of fatty acid beta‐oxidation,” and “mucus secretion.” By contrast, Epi (MACC1) showed malignant state signatures, including “positive regulation of cell division,” “regulation of actin cytoskeleton organization,” “PI3K‐Akt signaling pathway,” “Rap1 signaling pathway,” “Ras signaling pathway,” and “MAPK signaling pathway.” To validate these classifications, we performed GEP analysis of epithelial cells by cNMF and identified six GEPs (Figure S2D). GEP3, GEP4, and GEP6 corresponded to Epi (BNIP3), enriched for “Glycolysis/Gluconeogenesis” and “HIF‐1 signaling pathway;” GEP2 aligned with Epi (IGFBP7), enriched for “ECM‐receptor interaction” and “Cell adhesion molecules;” and GEP1 and GEP5 corresponded to Epi (TFF3) and Epi (MACC1), enriched for “regulation of actin cytoskeleton” and “mucin type O‐glycan biosynthesis” (Figure S2D,E). This concordance between clustering‐based annotation and cNMF programs supported the robustness of these epithelial state assignments.

Notably, Epi (TFF3) showed enrichment of lipid metabolism‐related pathways, consistent with the lipophilic colonization pattern of PMP observed during surgery. Fat‐rich organs, including the greater omentum, mesentery, and epiploic appendages, are the major colonization sites of MT, and after extensive tumor involvement, only small amounts of residual fat remained (Figure S2F), which potentially supports that PMP may possess active lipid metabolic capacity. To evaluate metabolic features, we extracted Reactome metabolism‐related pathways and quantified metabolic scores with scMetabolism. At the subcluster level, Epi (TFF3) showed relatively high overall metabolic activity, and by tissue origin, its activity was significantly higher in MT than in PT, particularly for lipid metabolism pathways; Epi (MACC1) showed a similar trend (Figure 2E,F). Additionally, FABP1, involved in fatty acid uptake, trafficking, and β‐oxidation [39], was highly expressed in epithelial cells (Figure 2B; Figure S2A), and mIHC confirmed colocalization of FABP1 (red) with PanCK (green). Importantly, the enrichment of FABP1 in epithelial cells was significantly higher in MT than in PT (Figure 2G), further supporting enhanced lipid metabolic reprogramming in metastatic PMP epithelial cells.

Collectively, these results identify four functionally distinct epithelial subclusters in PMP and support lipid metabolism‐associated reprogramming as a prominent feature, especially in MT‐associated epithelial states.

### 3.3 Identification of Malignant‐ and Mucus Secretion‐Associated Epithelial Subclusters by InferCNV and Pseudotime Analyses

**FIGURE 3:**
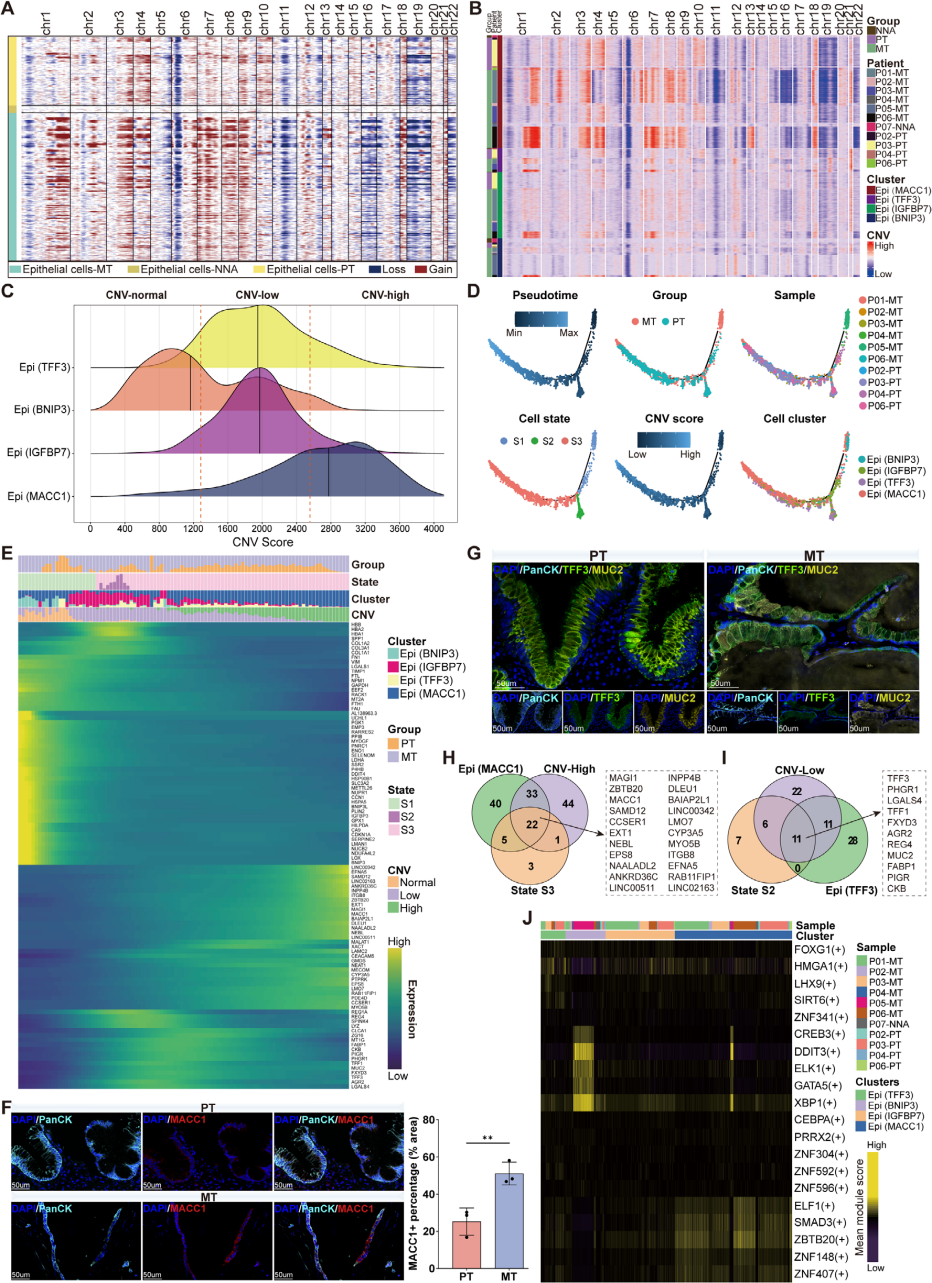
InferCNV and pseudotime analyses identify diverse tumor epithelial cell states. (A) InferCNV heatmaps showing large‐scale inferred CNV patterns in epithelial cells grouped by tissue origin. (B) Heatmap showing the distribution of inferred CNV events across epithelial subclusters, tissue origin, and sample. (C) Density distribution of inferred CNV scores across epithelial subclusters. Epithelial cells were stratified into CNV‐normal, CNV‐low, and CNV‐high groups according to predefined global thresholds. Red dashed lines indicate classification thresholds. (D) Pseudotime trajectory of epithelial cell is colored by pseudotime (top left), tissue origin (top middle), sample (top right), pseudotime‐associated state (bottom left), inferred CNV score (bottom middle), and epithelial subcluster (bottom right). (E) Heatmap of genes varying along the pseudotime trajectory. The bar chart above the heatmap shows the distribution of tissue origin, pseudotime‐associated state, epithelial subcluster, and CNV group. (F) mIHC showing the colocalization of DAPI (blue), PanCK (light blue), and MACC1 (red) in PT and MT samples. Scale bars, 50 µm. The accompanying bar plot shows the quantification results of three independent patient samples per group. Three representative fields were quantified per sample, and the averaged value for each sample was used for statistical analysis. Data are presented as mean ± SEM. Statistical analysis was performed using a two‐tailed Student's *t*‐test. ^**^ *p* < 0.01. (G) Representative mIHC images showing PanCK (light blue), TFF3 (green), and MUC2 (yellow) staining in PT and MT samples. (H, I) Venn diagrams showing the intersection of differentially expressed genes among Epi (MACC1) subcluster, CNV‐high group, and pseudotime state S3 (H), or among Epi (TFF3) subcluster, CNV‐low group, and pseudotime state S2 (I). (J) Heatmap displaying epithelial subcluster‐specific regulon activity inferred by SCENIC, with top annotations indicating sample identity and epithelial subcluster.

To characterize potentially malignant epithelial states, we inferred epithelial CNV patterns with inferCNV, using immune cells as references. NNA epithelial cells showed minimal inferred‐CNV burden, whereas widespread CNV events occurred in PT and MT, particularly on chr1, chr3, chr7, chr8, chr9, chr18, and chr21. Inferred‐CNV burden was highly heterogeneous across tissue sources and patients (Figure 3A,B).

To support genomic abnormalities in tumor samples, we analyzed WES data from MT of five patients with matched scRNA‐seq (P01, P02, P03, P05, and P06). The WES results showed that CNV patterns across major chromosomes were largely consistent with those inferred from scRNA‐seq (Figure S3A). In addition, multiple driver mutations were revealed, including KRAS, GNAS, BRAF, JAK1, SMARCA4, TP53, and APC, with RAS pathway‐related mutations particularly prominent (Figure S3B,C).

To classify epithelial cells by inferred‐CNV burden, we applied the predefined global Gaussian mixture model‐based framework to the pooled CNV accumulation scores. The fitted component means were 634.8, 1930.9, and 3201.5, giving global thresholds of 1282.9 and 2566.2 that separated cells into CNV‐normal, CNV‐low, and CNV‐high groups. The relative proportions of the three groups varied across samples (Figure 3C; Figure S4A,B). CNV‐high cells preferentially expressed malignant‐associated genes such as MACC1, ITGB8, and LAMB3, and mainly corresponded to Epi (MACC1). CNV‐low cells preferentially expressed mucus secretion, lipid metabolism, and stromal remodeling genes, including TFF3, MUC2, REG4, AGR2, FABP1, and IGFBP7, and mainly corresponded to Epi (TFF3) and Epi (IGFBP7). CNV‐normal cells highly expressed hypoxia‐adaptation genes such as BNIP3 and ENO2, and corresponded to Epi (BNIP3) (Figures S2A and S4C).

To explore transcriptomic ordering within the tumor epithelial compartment, we performed pseudotime analysis with Monocle2, identifying two major branches and three pseudotime‐associated states (S1–S3) (Figure 3D). State‐specific highly expressed genes in S1, S2, and S3 broadly corresponded to CNV‐normal, CNV‐low, and CNV‐high, respectively (Figure S4C,D). PT‐ and MT‐derived epithelial cells were distributed across all three states, though their relative representation varied. Epi (BNIP3) localized near the pseudotime origin and was enriched in the CNV‐normal group, consistent with a benign‐like state, whereas Epi (MACC1) was enriched in branch‐terminal regions with the highest inferred‐CNV burden, consistent with a malignant‐like state. Epi (TFF3) and Epi (IGFBP7) occupied intermediate regions, consistent with intermediate states (Figure 3D).

We next integrated epithelial subclusters, inferred‐CNV burden, tissue origin, and pseudotime states to characterize PMP epithelial programs (Figure 3E). Mucus secretion‐related genes (REG4, MUC2, TFF3, and AGR2) and the lipid metabolism gene FABP1 were most highly expressed in regions corresponding to S2, Epi (TFF3), and CNV‐low, whereas malignancy‐associated genes such as MACC1 and ITGB8 were highest in regions corresponding to S3, Epi (MACC1), and CNV‐high. Because excessive mucus secretion and abdominopelvic dissemination are central features of PMP, Epi (TFF3) and Epi (MACC1) are the primary subclusters for subsequent study. mIHC confirmed MACC1 expression in PanCK‐positive epithelial structures in PT and MT and showed TFF3 and MUC2 patterns in epithelial cells (Figure 3F,G). MACC1 has been extensively implicated in proliferation, invasion, metastasis, and immune evasion across tumor types [40]. We observed that the enrichment of MACC1 in epithelial cells was significantly higher in MT than in PT (Figure 3F), highlighting its potential role in malignant MT behavior. MUC2 staining in PT was largely cytoplasmic, whereas in MT it showed prominent apical aggregation and extracellular deposition (Figure 3G; Figure S2C).

We next identified key gene sets for mucus secretion and malignant state by intersecting differentially expressed gene subsets defined by S2/Epi (TFF3)/CNV‐low and S3/Epi (MACC1)/CNV‐high, respectively (Figure 3H,I). Several malignancy‐associated genes tended toward higher expression in MT, whereas mucus secretion‐associated genes showed more variable patterns across samples (Figure S5A,B), and were subsequently validated in an independent bulk RNA‐seq cohort (*n* = 57) (Figure S5C). These genes may serve as candidate features for molecular stratification and exploratory biomarkers in PMP. Finally, SCENIC identified subcluster‐specific transcription factors, including DDIT3 for Epi (BNIP3), CEBPA for Epi (IGFBP7), LHX9 for Epi (TFF3), and ZBTB20 for Epi (MACC1) (Figure 3J; Figure S6), providing clues for mechanistic studies.

Collectively, integrative pseudotime and inferCNV analysis provided an exploratory framework for organizing transcriptional heterogeneity within PMP tumor epithelial cells and identified Epi (TFF3) and Epi (MACC1) as key subclusters associated with mucus secretion‐related and malignant‐like programs, respectively.

### 3.4 Immune‐Excluded Phenotype Associated with a Collagen‐Rich Peritumoral Stromal Barrier

**FIGURE 4:**
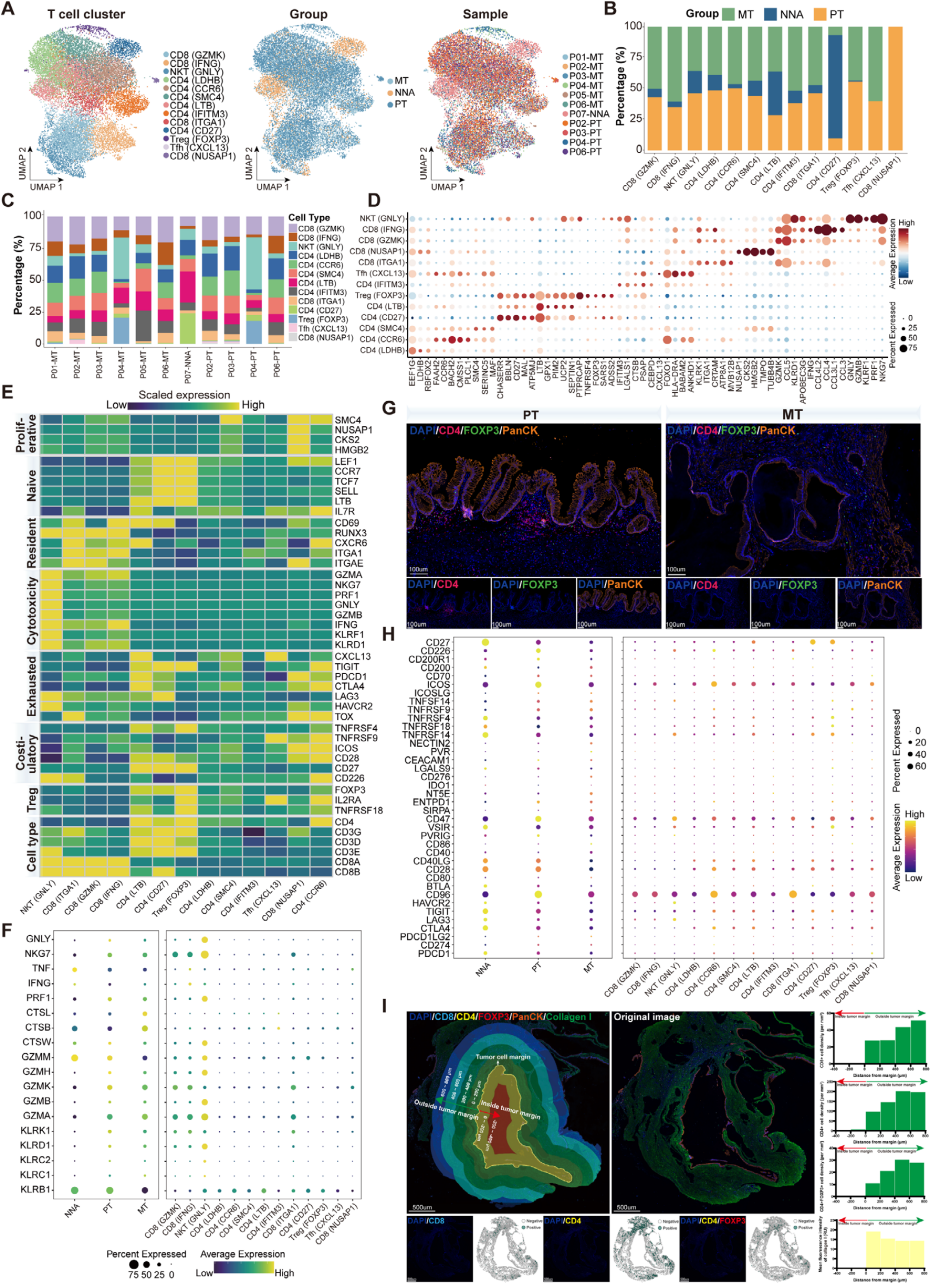
Transcriptional characteristics of T cells and stromal barrier‐associated immune exclusion. (A) UMAP plots of T cells colored by T cell subcluster (left), tissue origin (middle), and sample (right). (B, C) Stacked bar plots showing the relative proportion of tissue origins across T cell subclusters (B), and the relative proportions of T cell subclusters across individual samples (C). (D) Dot plot showing representative marker genes for each T cell subcluster. (E). Heatmap illustrating the relative expression patterns of predefined functional gene signatures across T cell subclusters, including proliferative, naive, resident, cytotoxicity, exhausted, co‐stimulator, Treg, and cell type‐associated signatures. (F) Dot plot showing the relative expression levels of cytotoxicity‐related genes across tissue origins (left) and T cell subclusters (right). (G) mIHC showing colocalization of DAPI (blue), CD4 (red), FOXP3 (green), and PanCK (orange) in PT and MT. Scale bars, 100 µm. (H) Dot plot showing the relative expression levels of ICGs across tissue origins (left) and T cell subclusters (right). (I) mIHC of MT showing colocalization of DAPI (blue), CD8 (light blue), CD4 (yellow), FOXP3 (red), PanCK (orange), and Collagen I (green). Using the PanCK‐positive epithelial margin as the reference interface, tissue regions were classified as inside or outside the tumor margin and further divided into consecutive 200 µm distance bins (upper left). Original images and corresponding spatial maps of CD8^+^ T cells, CD4^+^ T cells, and FOXP3^+^ Treg cells are shown in the lower panels. Scale bars, 500 µm. Bar plots on the right summarize the densities of T cell subclusters, as well as the mean fluorescence intensity of Collagen I, across consecutive 200 µm distance bins relative to the tumor margin. Analyses were performed from three independent patient samples (one field per patient; three fields in total).

Given the central role of T cells in antitumor immunity, we characterized their composition and transcriptional features to better understand the PMP immune microenvironment. Unsupervised clustering of 19,226 T cells identified 13 subclusters: eight CD4^+^ T cell, four CD8^+^ T cell, and one NKT cell subcluster (Figure 4A). By tissue distribution, Tfh (CXCL13) was detected exclusively in tumor tissues, CD4 (CD27) predominated in the NNA reference sample, and CD8 (NUSAP1) was specifically enriched in PT (Figure 4B,C).

NKT (GNLY) highly expressed cytotoxicity‐related genes, including GNLY, NKG7, GZMB, and KLRF1 (Figure 4D,E); at the sample level, these genes tended to be lower in MT than in PT (Figure 4F; Figure S7A). Among CD8^+^ T cell subclusters, CD8 (NUSAP1) highly expressed proliferation‐associated genes (SMC4, NUSAP1, CKS2, and HMGB2) with limited cytotoxic features, while the remaining subclusters, CD8 (ITGA1), CD8 (GZMK), and CD8 (IFNG), showed resident‐like features and similarly limited cytotoxicity (Figure 4D–F). Within the CD4^+^ compartment, CD4 (CCR6), CD4 (LTB), and CD4 (CD27) showed representation of the predefined co‐stimulatory‐, exhaustion‐, and naive‐associated gene signatures. Treg (FOXP3) showed low detectable expression of selected exhaustion‐associated genes, including PDCD1, CTLA4, and LAG3 (Figure 4D,E). Given the inherent sparsity of single‐cell data, these patterns should not be overinterpreted as definitive functional states. mIHC confirmed Treg (FOXP3) within stromal regions of both PT and MT (Figure 4G): in MT, Tregs were predominantly stromal and spatially separated from tumor epithelial cells, whereas in PT, some Tregs lay close to epithelial structures, suggesting potential interaction.

Because immune checkpoint molecules are major regulators of T‐cell activity [41], we examined immune checkpoint gene (ICG) expression in T cells across tissue origins and subclusters. ICGs were detected at generally low levels in PMP tumor‐associated T cells, with a descriptive tendency toward sparser expression in MT (Figure 4H), consistent with a restrained activation state. This may relate to the distinctive architecture of MT, where tumor epithelial cells line the inner surface of cystic spaces, the luminal side enriched in mucus, and the outer side surrounded by a dense stromal compartment (Figure S7B). Such an organization may hinder contact between T cells and tumor epithelial cells, limiting effective antigenic stimulation.

To more rigorously assess T‐cell spatial organization in MT, we performed additional mIHC‐based spatial analyses. The panel (CD4, CD8, FOXP3, PanCK, and Collagen I) allowed simultaneous visualization of T cell subsets, tumor epithelial cells, and stromal barrier. Using the PanCK‐positive tumor epithelial lining the inner cystic wall as the reference margin, each lesion was divided into regions inside and outside the tumor margin, then into consecutive 200 µm distance bins (Figure 4I). We then quantified the densities of CD8^+^ T cells, CD4^+^ T cells, and CD4^+^ FOXP3^+^ T cells (Tregs) across these spatial intervals. All three T cell populations were largely restricted to the stroma outside the tumor margin and rarely detected within the epithelial boundary; moreover, on the stromal side, T cell density exhibited a trend of increasing with the distance from the margin.

To characterize the stromal barrier adjacent to the tumor epithelium, we quantified collagen I mean fluorescence intensity across the same bins. Collagen I peaked in the peritumoral stroma immediately adjacent to the tumor boundary and tended to decrease with increasing distance. Accordingly, stroma closest to the boundary showed the highest collagen signal and lowest T cell density, whereas more distant stroma showed lower collagen and higher T cell density (Figure 4I).

Collectively, MT exhibits a stroma‐confined T cell distribution associated with a collagen‐rich peritumoral stromal barrier, consistent with an immune‐excluded phenotype.

### 3.5 Heterogeneity of Tumor‐Associated Fibroblast Subclusters and Their Potential Role in Stromal Remodeling

**FIGURE 5:**
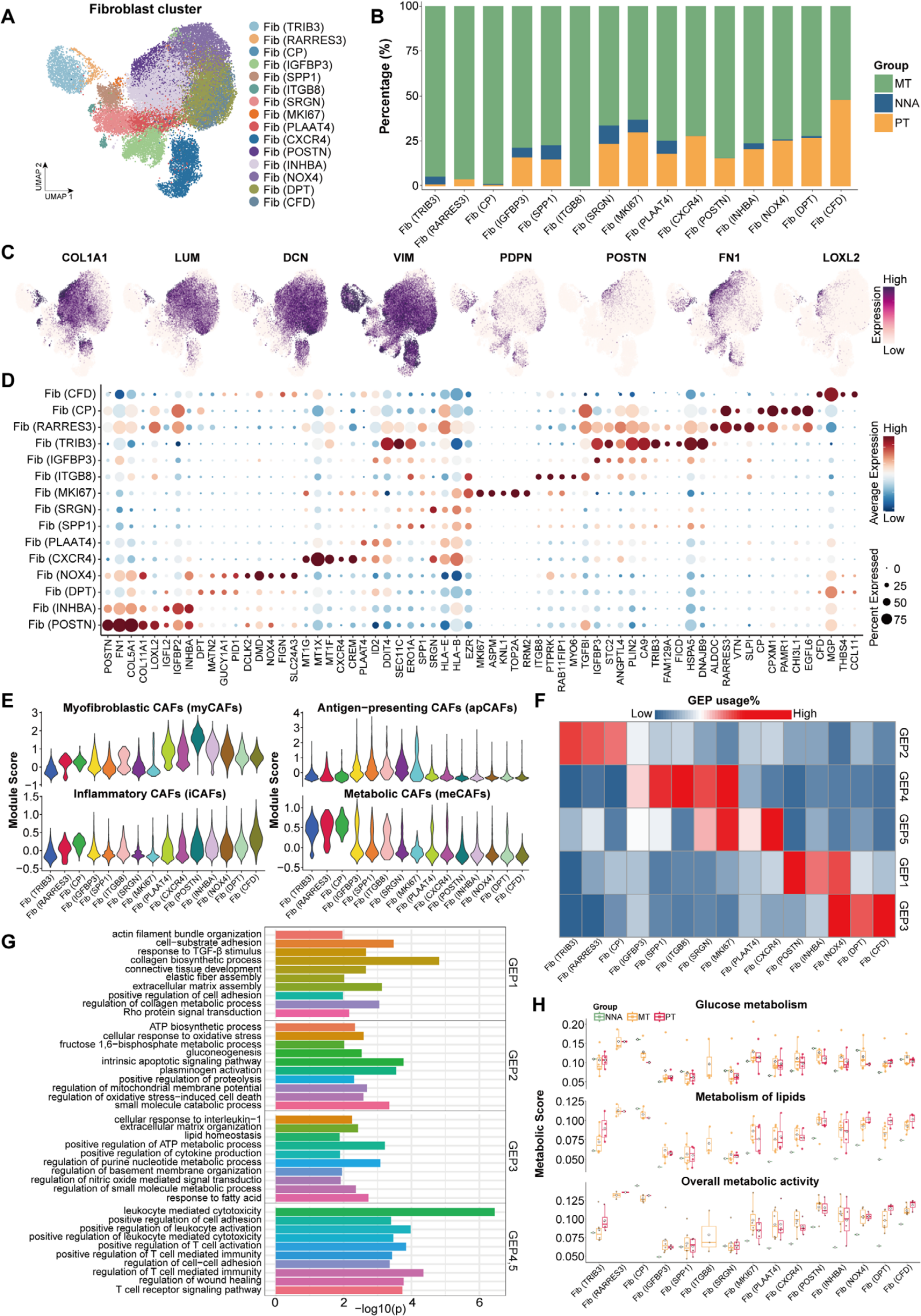
Transcriptional characteristics of fibroblasts in the tumor microenvironment. (A) UMAP plot showing fibroblast subclusters. (B) Stacked bar plot showing the relative proportion of tissue origins across fibroblast subclusters. (C) UMAP plots illustrating expression of selected CAF‐associated and matrix‐remodeling genes in fibroblasts. (D) Dot plot displaying representative marker genes for each fibroblast subcluster. (E) Violin plots showing module scores of fibroblast subclusters for myCAFs, iCAFs, apCAFs, and meCAFs programs. (F) Heatmap showing the usage patterns of five GEPs identified by cNMF across fibroblast subclusters. (G) Top 10 significantly enriched biological processes among differentially expressed genes for each GEP based on GO enrichment analysis. (H) Box plots showing glucose metabolism activity (top), lipid metabolism activity (middle), and overall metabolic activity (bottom) of fibroblast subclusters across tissue origins. Data are presented as median with IQR. Statistical analysis of the comparison between the PT (*n* = 4) and MT (*n* = 6) groups was performed using the Wilcoxon rank‐sum test.

During CRS, extensive stromal deposition and sclerosis in MT markedly increase surgical difficulty, underscoring the clinical significance of systematically characterizing fibroblast functions within the PMP tumor microenvironment. We identified 15 subclusters among 31,809 fibroblasts (Figure 5A). By tissue distribution, Fib (INHBA), Fib (PLAAT4), Fib (SRGN), and Fib (IGFBP3) predominated in NNA, whereas Fib (SPP1), Fib (MKI67), Fib (CXCR4), Fib (POSTN), Fib (NOX4), Fib (DPT), and Fib (CFD) were mainly in PT and MT (Figure 5B; Figure S8A,B).

These fibroblast subclusters broadly expressed cancer‐associated fibroblast (CAF) markers, including COL1A1, LUM, DCN, VIM, and PDPN (Figure 5C). CAFs are known to promote tumor fibrosis, vascular abnormalities, proliferation, and invasion through extracellular matrix deposition and remodeling and secretion of factors such as transforming growth factor‐beta (TGF‐β) and to foster an immunosuppressive microenvironment [42, 43]. To characterize CAF states in PMP, we built CAF‐related functional modules from known marker genes [44, 45] and classified fibroblast subclusters according to module scores. Fib (POSTN), Fib (INHBA), Fib (DPT), Fib (NOX4), Fib (PLAAT4), and Fib (CXCR4) were classified as myofibroblastic CAFs (myCAFs), with high TAGLN and ACTA2 expression. Fib (SPP1), Fib (SRGN), Fib (IGFBP3), Fib (ITGB8), and Fib (MKI67) were antigen‐presenting CAFs (apCAFs), with high antigen‐presentation genes including HLA‐DRA. Fib (CFD), expressing high CFD, was classified as inflammatory CAFs (iCAFs), whereas Fib (TRIB3), Fib (RARRES3), and Fib (CP) scored relatively high in the metabolic CAF (meCAF) module (Figure 5D,E).

To characterize these subclusters, we applied cNMF to decompose fibroblasts into five GEPs, followed by enrichment‐based functional annotation (Figure 5F,G; Figure S8C). GEP2 was associated with “gluconeogenesis,” “HIF‐1 signaling pathway,” “ATP biosynthetic process,” and “cellular response to oxidative stress,” correlating with energy metabolism and the meCAF subclusters. GEP4 and GEP5 were associated with immune‐regulatory pathways, including “antigen processing and presentation,” “regulation of T cell‐mediated immunity,” and “positive regulation of leukocyte activation,” corresponding to apCAFs. GEP3 was associated with “extracellular matrix organization,” “ECM‐receptor interaction,” “cellular response to interleukin‐1,” and “positive regulation of cytokine production,” mainly in Fib (NOX4), Fib (DPT), and Fib (CFD). Notably, GEP1 was highly enriched in matrix production, assembly, and stromal sclerosis pathways, including “collagen biosynthetic process,” “cell‐substrate adhesion,” “cellular response to transforming growth factor‐beta stimulus,” and “elastic fiber assembly,” and was primarily represented by Fib (POSTN), Fib (INHBA), and Fib (NOX4). Among them, Fib (POSTN) showed the major contribution to GEP1 and highly expressed matrix‐remodeling genes such as POSTN, FN1, and LOXL2 (Figure 5C,F), which promote matrix deposition, alignment, and crosslinking, thereby increasing stromal stiffness, altering tumor texture, and impeding immune cell infiltration and drug penetration [46, 47, 48]. These findings nominate Fib (POSTN) as a candidate contributor to stromal remodeling and stiffening in PMP. Characterizing the metabolic landscape, we found broad lipid metabolism‐related activity across several fibroblast states (Figure 5H; Figure S8D): besides the high activity of meCAFs, myCAFs also showed relatively high scores, particularly Fib (POSTN), consistent with the energy demand of large‐scale stromal remodeling.

Collectively, our analysis delineated a highly heterogeneous fibroblast landscape in PMP and identified Fib (POSTN) as a prominent myCAF subcluster with strong stromal remodeling and metabolic activity, exhibiting a pronounced profibrotic program that may contribute to stromal sclerosis in PMP.

### 3.6 Lipid‐Associated Macrophages Are Associated with Immunosuppressive Chemokine Expression in PMP

**FIGURE 6:**
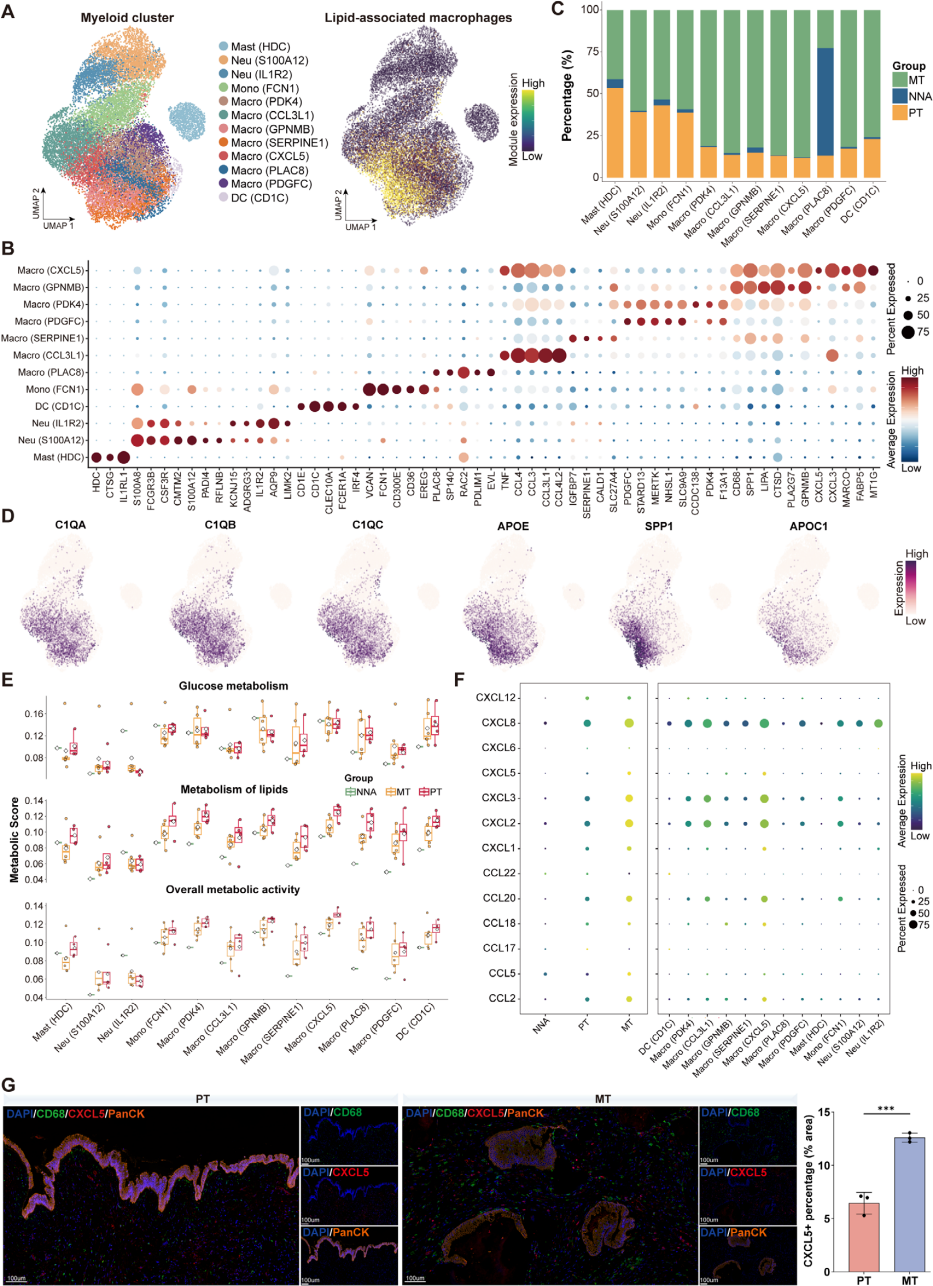
Transcriptional characteristics of myeloid cells in the tumor microenvironment. (A) UMAP plots of myeloid cells colored by cell subcluster (left) and lipid‐associated macrophage module expression (right). (B) Dot plot displaying representative marker genes for each myeloid cell subcluster. (C) Stacked bar plot showing the relative proportion of tissue origins across myeloid cell subclusters. (D) UMAP plots illustrating the expression of selected tumor‐associated macrophage and lipid metabolism‐related genes in myeloid cells. (E) Box plots showing glucose metabolism activity (top), lipid metabolism activity (middle), and overall metabolism activity (bottom) of myeloid cell subclusters across tissue origins. Data are presented as median with IQR. Statistical analysis of the comparison between the PT (*n* = 4) and MT (*n* = 6) groups was performed using the Wilcoxon rank‐sum test. (F) Dot plot showing the relative expression levels of selected chemokines across tissue origins (left) and myeloid cell subclusters (right). (G) mIHC showing colocalization of DAPI (blue), CD68 (green), CXCL5 (red), and PanCK (orange) in PT and MT. Scale bars, 100 µm. The accompanying bar plot shows the quantification results of three independent patient samples per group. Three representative fields were quantified per sample, and the averaged value for each sample was used for statistical analysis. Data are presented as mean ± SEM. Statistical analysis was performed using a two‐tailed Student's *t*‐test. ^***^ *p* < 0.001.

Given that myeloid cells are also a key component of the tumor immune microenvironment, we proceeded to characterize the transcriptional states of myeloid cell subclusters. Unsupervised clustering of myeloid cells identified 12 subclusters (Figure 6A): one mast cell subcluster, Mast (HDC), supported by mast cell‐associated markers IL1RL1, CTSG, and HDC; two neutrophil subclusters, Neu (S100A12) and Neu (IL1R2), supported by canonical neutrophil‐associated markers including FCGR3B, CSF3R, and S100A8; one monocyte subcluster, Mono (FCN1), supported by FCN1 and VCAN; one dendritic‐cell subcluster, DC (CD1C), supported by CD1C; and seven macrophage subclusters supported by pan‐macrophage markers, including CD68 (Figure 6B). Macro (PLAC8) predominated in NNA, while the remaining macrophage subclusters were mainly in PT and MT (Figure 6C; Figure S9A,B).

Macrophage subclusters broadly expressed pan‐tumor‐associated macrophage genes, including C1QA, C1QB, and C1QC (Figure 6D). Besides the conventional “M1‐like” subcluster Macro (CCL3L1) and the “M2‐like” subclusters Macro (SERPINE1) and Macro (PDGFC), we identified a tissue‐resident subcluster, Macro (PLAC8), with high SP140 and PLAC8 and relatively higher RAC2. We also identified three lipid‐associated macrophage (LAM) subclusters, Macro (PDK4), Macro (GPNMB), and Macro (CXCL5), expressing lipid metabolism genes such as SPP1, APOC1, and APOE (Figure 6A,B,D). mIHC confirmed colocalization of APOE (red) and CD68 (pink) in PT and MT (Figure S9C), supporting LAMs in the PMP microenvironment.

LAMs reportedly exhibit active lipid metabolism that may support tumor progression under metabolic stress and foster an immunosuppressive microenvironment [49]. Evaluating metabolic activity across myeloid subclusters, the LAM subclusters Macro (PDK4), Macro (GPNMB), and Macro (CXCL5) tended to show relatively high overall metabolic scores, with similar patterns across lipid metabolism pathways (Figure 6E; Figure S9D). Examining chemokine expression, CCL5 was significantly higher in MT than in PT, whereas CCL2, CCL18, CCL20, CXCL1, CXCL2, CXCL3, CXCL5, and CXCL8 showed a descriptive trend toward higher MT expression, with Macro (CXCL5) expressing relatively high levels of several of these chemokines among the myeloid subclusters (Figure 6F; Figure S9E). These chemokines have been implicated in recruiting Tregs and myeloid‐derived suppressor cells and in suppressing effector immune cells, including CD8^+^ T cells and NK cells [50], supporting Macro (CXCL5) as a potential contributor to local immunosuppressive signaling in MT. mIHC further showed colocalization of CD68 (green) with CXCL5 (red) in tumor tissues, and confirmed that the enrichment of CXCL5 in macrophages was significantly higher in MT than in PT (Figure 6G).

Collectively, these findings identify Macro (CXCL5) as a LAM subcluster enriched in MT with relatively elevated metabolic and immunosuppressive chemokine‐expression trends, supporting its potential association with immunosuppressive signaling in the PMP microenvironment.

### 3.7 A Candidate Pro‐Angiogenic Network Mediated by VEGFA‐VEGFR2 Signaling

A major pathological feature of PMP is persistent mucus production, especially in MT. Because this process requires vascular support, we next examined angiogenesis‐related cellular programs in PMP.

**FIGURE 7:**
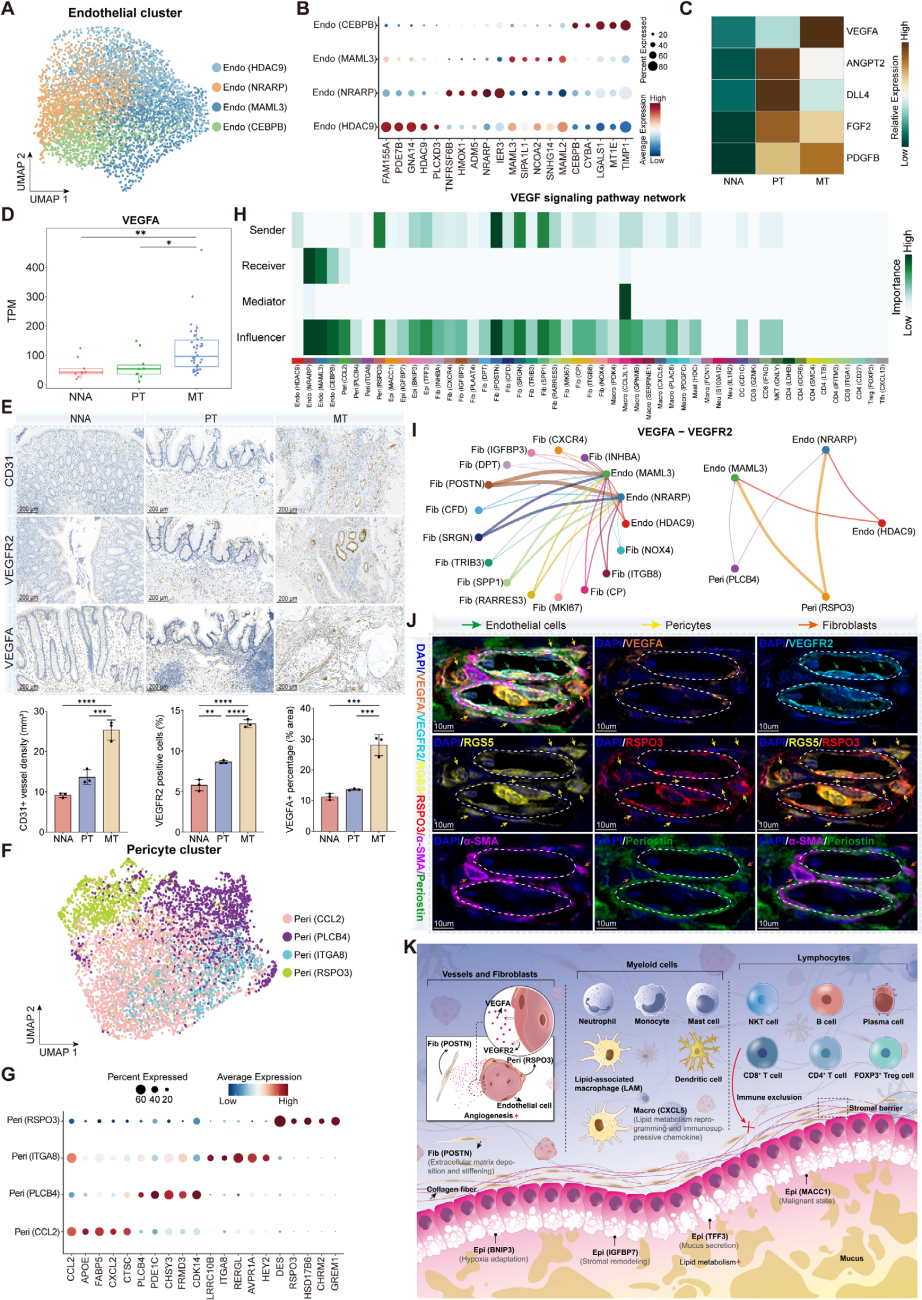
Angiogenic network in the tumor microenvironment. (A) UMAP plot showing endothelial cell subclusters. (B) Dot plot illustrating representative marker genes for each endothelial cell subcluster. (C) Heatmap showing the relative expression levels of selected pro‐angiogenic factors across tissue origins in scRNA‐seq dataset. (D) Box plots showing VEGFA expression across tissue origins in an independent bulk RNA‐seq cohort (NNA = 9, PT = 10, and MT = 38). Data are presented as median with IQR. Statistical significance was assessed using the Kruskal‐Wallis test followed by Dunn's multiple‐comparisons test. ^*^ *p* < 0.05, ^**^ *p* < 0.01. (E) The upper panel shows representative IHC images of CD31, VEGFR2, and VEGFA in NNA, PT, and MT samples. Scale bars, 200 µm. The lower panel shows the corresponding quantitative analysis of three independent patient samples per group, with three representative fields quantified per sample and the averaged value for each sample used for statistical analysis. The NNA group comprised the single NNA specimen included in the scRNA‐seq cohort plus two additional NNA specimens collected independently. Data are presented as mean ± SEM. Statistical analysis was performed using one‐way ANOVA followed by Tukey's multiple‐comparisons test. ^**^ *p* < 0.01, ^***^ *p* < 0.001, ^****^ *p* < 0.0001. (F) UMAP plot showing pericyte subclusters. (G) Dot plot showing representative marker genes for each pericyte subcluster. (H) CellChat analysis showing the VEGF signaling pathway network in MT. (I) Circle plot depicting the strength of VEGFA‐VEGFR2 ligand‐receptor interactions between fibroblast and endothelial cell subclusters (left), and between pericyte and endothelial cell subclusters (right) based on CellChat analysis. (J) Representative mIHC images of MT showing colocalization of DAPI (blue), VEGFA (orange), VEGFR2 (light blue), RGS5 (yellow), RSPO3 (red), α‐SMA (purple), and Periostin (green). The white dashed line indicates the vascular boundary. VEGFR2^+^ endothelial cells (green arrow) line the luminal side of the vessel, while VEGFA‐expressing RGS5^+^ RSPO3^+^ pericytes (yellow arrow) and α‐SMA^+^ POSTN^+^ fibroblasts (orange arrow) are located adjacent to the vessels. Scale bars, 10 µm. (K) Schematic model summarizing the multicellular ecosystem of metastatic PMP lesions.

Our scRNA‐seq data identified 5,428 endothelial cells, divided into four subclusters: Endo (HDAC9), Endo (NRARP), Endo (MAML3), and Endo (CEBPB), each with distinct characteristic gene profiles (Figure 7A,B). To assess pro‐angiogenic signaling, we examined key angiogenesis‐related genes across tissue types. In scRNA‐seq, VEGFA trended higher in MT than in PT (Figure 7C; Figure S10A), while in the independent bulk RNA‐seq cohort, VEGFA was significantly higher in MT than in PT and NNA among these pro‐angiogenic factors (Figure 7D; Figure S10B). In addition, IHC revealed that vascular density and the expression levels of VEGFA and VEGFR2 were significantly higher in MT than in PT and NNA samples (Figure 7E). Given that VEGFA‐VEGFR2 signaling is central to physiological and tumor angiogenesis [51], these findings are consistent with enhanced angiogenic activity in MT.

Because pericytes are critical regulators of microvessel stability, permeability, and perfusion [52], we characterized them, identifying four subclusters: Peri (CCL2), Peri (PLCB4), Peri (ITGA8), and Peri (RSPO3), each with distinct characteristic genes (Figure 7F,G). To define the core components of the angiogenic module, we used CellChat to visualize intercellular communication through the VEGF pathway in MT. We observed extensive VEGF‐related interactions between endothelial cells and multiple ligand‐producing populations, including pericytes, epithelial cells, fibroblasts, macrophages, dendritic cells, and T cells, among which Fib (POSTN) and Peri (RSPO3) emerged as prominent candidate partners of endothelial cells (Figure 7H); the VEGFA‐VEGFR2 signaling supported a similar interaction pattern (Figure 7I). mIHC showed VEGFR2^+^ endothelial cells lined the luminal side of the vessel, while VEGFA‐expressing RGS5^+^ RSPO3^+^ pericytes and α‐SMA^+^ POSTN^+^ fibroblasts were located adjacent to the vessels (Figure 7J), a spatial arrangement compatible with VEGFA‐VEGFR2‐associated signaling and a cooperative pro‐angiogenic role in MT.

Collectively, these findings are consistent with enhanced angiogenic activity in MT and delineate a spatially organized, functionally coordinated candidate pro‐angiogenic network in which Fib (POSTN) and Peri (RSPO3) cooperate with endothelial cells through VEGFA‐VEGFR2 signaling to drive active neovascularization.

Based on these findings, we constructed a schematic diagram illustrating the characteristics of the PMP metastatic‐lesion microenvironment (Figure 7K).

### 3.8 Clinical Observations Suggest Potential Disease Control with Anti‐Angiogenic Therapy in Recurrent PMP

As described above, MT showed molecular and histologic features of active angiogenesis. To explore the potential clinical translatability, we retrospectively reviewed the clinical course of three recurrent PMP patients treated with anlotinib (a VEGFR2 inhibitor).

**FIGURE 8:**
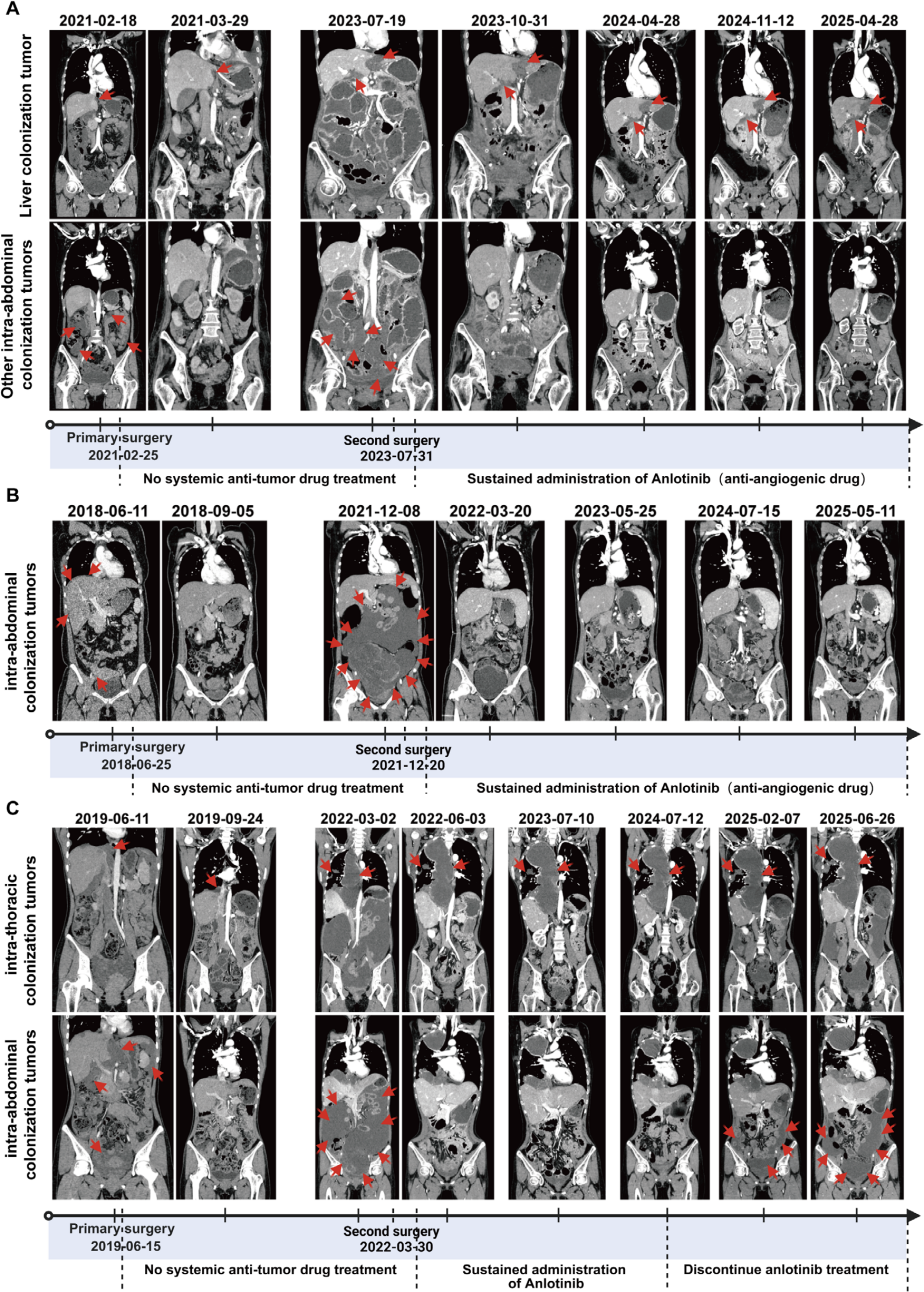
Clinical course of three patients with recurrent PMP treated with anti‐angiogenic therapy. (A‐C) Treatment timelines and representative CT images from three patients with recurrent PMP who received anlotinib after secondary CRS. In patients 1 and 2 (A and B), sustained anlotinib administration after secondary surgery was associated with prolonged radiological disease control until the last follow‐up. In patient 3 (C), the disease remained radiologically stable during anlotinib treatment but progressed after treatment discontinuation. Red arrows indicate low‐density metastatic lesions of PMP.

All patients developed recurrence 2–3 years after initial CRS and underwent a second CRS at our institution. Two patients began anlotinib treatment after the second CRS and remained free of overt progression to the last follow‐up, with progression‐free survival already exceeding the interval to first recurrence after initial CRS (Figure 8A,B). The third patient also started anlotinib after the second CRS, with radiology showing overall stable disease; however, after discontinuing anlotinib at 2 years, obvious progression was detected 7 months later (Figure 8C).

Collectively, these preliminary clinical observations are consistent with a potential role for VEGFR2‐targeted anti‐angiogenic therapy in delaying progression in recurrent PMP, though formal validation will require larger prospective studies.

## 4 Discussion

This study utilized high‐resolution single‐cell transcriptomics to characterize major cellular populations in PMP and analyze the diversity, metabolic features, and functional programs of tumor epithelial and microenvironment cells. We identified the mucus secretion‐associated Epi (TFF3) subcluster, the malignant‐associated Epi (MACC1) subcluster, and Fib (POSTN) subcluster with a potential role in stromal remodeling. In metastatic lesions, we observed patterns consistent with limited T‐cell access to epithelial structures, Treg infiltration, and inhibitory chemokine expression in Macro (CXCL5). In addition, we also identified widespread lipid metabolism‐related activity and a candidate pro‐angiogenic network involving Fib (POSTN), Peri (RSPO3), and endothelial cells, potentially mediated by VEGFA‐VEGFR2 signaling and preliminarily supported by clinical observations from three anlotinib‐treated patients.

A notable feature of PMP is that, despite the scarcity of epithelial cells, it was capable of producing massive amounts of mucus and causing substantial clinical symptoms, largely through highly glycosylated MUC2 secreted by tumor epithelial cells. The PTS domain of MUC2, rich in proline, threonine, and serine, carries extensive O‐linked glycans whose hydrophilic properties retain large amounts of water and dramatically expand the gel volume [53], and the resulting mucinous barrier around tumor cells substantially limits the penetration and efficacy of antitumor drugs. We identified the mucus secretion‐associated Epi (TFF3) subcluster, which highly expresses goblet cell markers such as TFF3, AGR2, and MUC2, consistent with the goblet cell identity of PMP epithelium established by Ayala et al. [26]. Strategies that reduce mucus production or degrade accumulated mucus, therefore, appear promising as potential therapeutic approaches in PMP. Morris and colleagues proposed a mucolytic strategy combining bromelain and N‐acetylcysteine (BromAc) to dissolve and clear existing mucus; in a multicenter prospective phase I trial, it achieved a 73.2% objective response rate at the treatment site [54], and more recent evidence further supports BromAc efficacy and safety in unresectable PMP [55, 56], underscoring mucolytic therapy as an emerging direction.

We observed a KRAS mutation frequency of 60%, broadly consistent with previous PMP genomic studies [16]. KRAS mutations drive tumor cell proliferation and survival mainly through constitutive RAS‐MAPK and PI3K‐AKT‐mTOR activation, and in mucinous tumors, they are closely linked to a hypersecretory mucus phenotype [57, 58]. The MEK inhibitor RDEA119 suppressed tumor growth in PMP xenografts and reduced MUC2 expression by decreasing AP1 binding to the MUC2 promoter [59]. Wei et al. [60] found that fatty acid synthase inactivation in murine colonic epithelium inhibited N‐terminal palmitoylation of MUC2, preventing its secretion and function. Dilly et al. [57] showed that the PI3K inhibitor pictilisib downregulated fatty acid synthase and, combined with the MEK inhibitor trametinib, both suppressed progression of KRAS‐mutant colon and appendiceal mucinous adenocarcinomas and reduced MUC2 expression, secretion, and palmitoylation. In other solid tumors, dual MEK‐PI3K inhibition has shown encouraging response and disease‐control rates [61, 62]. Our data also identified high‐frequency mutations in RAS pathway‐associated genes, and the malignant‐associated Epi (MACC1) subcluster showed high enrichment of PI3K‐AKT, RAS, and MAPK signaling pathways, together supporting the therapeutic potential of targeting RAS‐MAPK and PI3K‐AKT‐mTOR signaling in PMP. In addition, we also identified high‐frequency SMARCA4 mutations; because SMARCA4 inactivation can disrupt genome‐wide epigenetic regulation and promote malignant transformation [63], its role in PMP warrants further investigation.

Lipid metabolism reprogramming emerged as another prominent feature of PMP, particularly in MT, aligning with a recent scRNA‐seq study showing elevated fatty acid metabolic activity in metastatic lesions versus AMN [26]. Hanse et al. [64] similarly reported increased lipid metabolism‐related gene expression in PMP by untargeted metabolomics, supporting metabolic reprogramming as a recurrent feature. Our single‐cell analysis extends this by identifying a broadly distributed lipid metabolism‐associated ecosystem in MT spanning tumor cells, particularly Epi (TFF3), and microenvironmental populations including meCAFs, myCAFs, and LAMs, which may explain the preferential implantation and growth of MT at lipid‐rich sites. Enhanced lipid metabolism reportedly promotes tumor progression and reinforces immunosuppressive cells such as Tregs and myeloid‐derived suppressor cells while impairing NK and CD8^+^ effector T cells [65]. Several preclinical studies have explored lipid‐targeting strategies: Feng et al. [66] showed that inhibiting the fatty acid transporter CD36 suppressed breast cancer growth and restored sensitivity to HER2‐targeted therapy in lapatinib‐resistant models, and FABP‐family inhibitors showed promising anti‐tumor activity [67, 68]. Collectively, combinatorial targeting of lipid metabolic pathways may be a valuable therapeutic strategy in PMP.

Although immunotherapy has advanced markedly in several solid tumors [69, 70, 71, 72], the immune landscape of PMP remains poorly defined, and no immunotherapy‐based agents are approved. Previous single‐cell analyses identified infiltrating lymphocytes in PMP [26, 27]. Our findings extend these by systematically characterizing the immunosuppressive microenvironment of PMP, particularly in metastatic lesions, where physical exclusion and functional suppression act in concert. Physically, myCAFs such as Fib (POSTN) showed strong TGF‐β responsiveness and may contribute to excessive stromal deposition and sclerosis, forming a dense barrier around tumor cells; mIHC‐based analyses showed that the collagen‐rich peritumoral stromal barrier may play a role in limiting T‐cell infiltration into the tumor epithelial compartment, consistent with features of an immune‐excluded phenotype. Functionally, Treg infiltration was observed, and Macro (CXCL5) tended to express relatively high levels of chemokines linked to immunosuppression [73, 74]. This excluded, suppressed microenvironment implies that monotherapy with immune checkpoint inhibitors, such as anti‐PD‐1 or anti‐PD‐L1 agents, may have limited efficacy, particularly given low ICG expression. More rational strategies may therefore require combinations that disrupt the stromal barrier to restore immune infiltration, for example, by targeting TGF‐β signaling and relieving functional immunosuppression by modulating LAMs and their chemokine axes, creating an accessible, activatable immune ecosystem for subsequent checkpoint blockade.

Another notable finding is the prominent pro‐angiogenic program associated with MT, in line with earlier Doppler ultrasound and microangiographic evidence from Dohan et al. [75]. We resolved this tissue‐level feature into a cellular and molecular framework, identifying a candidate pro‐angiogenic network of Fib (POSTN), Peri (RSPO3), and endothelial cells, potentially mediated through VEGFA‐VEGFR2 signaling. Preliminary follow‐up from three recurrent PMP patients suggested anlotinib may delay progression, though this clearly requires validation in larger prospective cohorts. Small case series have also reported that bevacizumab, targeting VEGFA, may benefit recurrent PMP [76, 77]. Together, these findings support further evaluation of anti‐angiogenic therapy as a component of systemic PMP treatment.

A few limitations should be acknowledged. First, the overall sample size was relatively small, a common challenge in PMP research; more importantly, the NNA reference cohort was especially limited, consisting of a single sample not from a healthy donor. Findings involving the NNA sample should therefore be interpreted cautiously as a descriptive reference only, not definitive disease‐versus‐normal comparisons. Future studies will require larger multicenter cohorts, expanded NNA or normal appendix reference sampling, and preferably matched designs to robustly define baseline cellular states and disease‐associated alterations. Second, in PMP samples with abundant mucus and low cellular density, ambient RNA contamination is likely; transcript‐level interpretations involving mucus and related secretory programs, and downstream pathway or ligand‐receptor analyses depending on such genes, should be interpreted with appropriate caution. Third, the lack of a well‐annotated clinical database with long‐term follow‐up and the absence of mature PMP disease models currently limit prognostic assessment, mechanistic dissection, and functional validation of candidate biomarkers and therapeutic targets, an important priority for future work.

## 5 Conclusions

In conclusion, our study delineates PMP at single‐cell resolution and characterizes its metastatic lesions as a coordinated multicellular ecosystem shaped by epithelial heterogeneity and microenvironmental reprogramming. By integrating epithelial states with stromal remodeling, lipid metabolic adaptation, immune suppression, and pro‐angiogenic networks, we provide a more comprehensive framework for the biological basis of PMP progression, while also offering a rationale and resource for mechanistic research and the development of novel or combination therapeutic strategies.

## Author Contributions

C.X.G., G.D.L., J.Y.Y., and W.W. conceived the project and designed the experiments. X.L., L.A., Z.M.L., and Y.C.L. collected all tissue samples and performed processing. L.A., Z.M.L., and Y.C.L. participated in clinical data collection and data uploading. X.L. and L.W. performed the primary bioinformatics analyses, with L.A., Z.C.C.C., T.R.Z., M.J.L., S.H.W., K.L., and J.J.C. assisting in relevant sections of the data analysis. X.L. participated in ethical review, pathological section staining experiments, and drafted the manuscript. C.X.G., G.D.L., J.Y.Y., and W.W. reviewed and revised the manuscript. All authors discussed and approved the final manuscript.

## Funding

This work was supported by the National Natural Science Foundation of China (82073943, 82373962 and 82450103 to J.Y.Y., 82370544 and 82570656 to W.W., 82404037 to G.D.L., 82574507 to C.X.G., 82204533 to J.J.C.), the Natural Science Foundation of Hunan Province (2023JJ40931 to J.J.C., 2023JJ30822 to C.X.G.), the Scientific Research Program of FuRong Laboratory (2025PT5001 to W.W.), Furong Laboratory Science and Technology Projects (2023SK2083‐2 to J.Y.Y.), the Major Research Project for High‐Level Health and Wellness Talents of Hunan Province (R2023042 to C.X.G.) and the Wisdom Accumulation and Talent Cultivation Project of the Third Xiangya Hospital of Central South University (YX202110 to C.X.G.).

## Ethics Approval and Consent to Participate

All procedures comply with relevant laws and institutional guidelines and have been approved by the Ethics Committee of Xiangya Hospital, Central South University (2024101296). This study respects the privacy rights of human subjects and has obtained their informed consent.

## Conflicts of Interest

The authors declare no conflict of interest.

## Supporting information




**Supporting File 1**: advs76790‐sup‐0001‐SuppMat.docx.

**Supporting File 2**: advs76790‐sup‐0002‐TableS1‐S2.xlsx.

## Data Availability

The single‐cell RNA‐sequencing, whole‐exome sequencing, and bulk RNA‐sequencing data used in this study have been uploaded to a public database (Human Genome Sequence Archive, accession number: HRA014174). Access is controlled and available from the corresponding author upon reasonable request.
