# Supplementary material for: Single‐Cell Transcriptomic Analysis of Tumor Heterogeneity and the Microenvironment in Pseudomyxoma Peritonei
Source: Adv Sci (Weinh). 2026 Jul 27:e23760. Online ahead of print. doi: 10.1002/advs.202523760 (PMC13403377; doi:10.1002/advs.202523760)
Supplement: Supplementary file 1 — Supporting File 1: advs76790‐sup‐0001‐SuppMat.docx. [file ADVS-9999-e23760-s002.docx]

Supplementary Information for

**Single-Cell Transcriptomic Analysis of Tumor Heterogeneity and the Microenvironment in Pseudomyxoma Peritonei**

Xi Li ^1, 2, 3, 11^, Lei Wang ^4^, Lei Ai ^1, 2, 3^, Zimeng Li ^1, 2, 3^, Zhaochangci Chen ^5^, Yuchen Luo ^1, 2, 3^, Tianran Zhang ^1, 2, 3^, Mengjia Liao ^1, 2, 3^, Songhao Wang ^1, 2, 3^, Jiajia Cui ^2, 6, 7, 8^, Ke Liu ^2, 6, 7, 8^, Chengxian Guo ^9, 10*^, Guodong Liu ^1, 2, 3*^, Jiye Yin ^2, 6, 7, 8, 11*^, Wei Wu ^1, 2, 3, 11*^

^1^Department of Geriatric Surgery, Xiangya Hospital, Central South University, Xiangya Road 87, Changsha, Hunan 410008, P. R. China

^2^National Clinical Research Center for Geriatric Disorders, 87 Xiangya Road, Changsha, Hunan 410008, P. R. China

^3^Department of General Surgery, Xiangya Hospital, Central South University, Changsha, Hunan 410008, P. R. China

^4^Liangzhu Laboratory, Zhejiang University, Hangzhou 310000, P. R. China

^5^Department of Ophthalmology, The Third Xiangya Hospital, Central South University, Changsha, Hunan 410013, P. R. China

^6^Department of Clinical Pharmacology, Xiangya Hospital, Central South University, Changsha, Hunan 410008, P. R. China

^7^Institute of Clinical Pharmacology, Central South University, Hunan Key Laboratory of Pharmacogenetics, Changsha, Hunan 410008, P. R. China

^8^Engineering Research Center of Applied Technology of Pharmacogenomics, Ministry of Education, 110 Xiangya Road, Changsha, Hunan 410000, P. R. China

^9^Center of Clinical Pharmacology, the Third Xiangya Hospital, Central South University, Changsha, Hunan 410013, P. R. China

^10^Xiangya School of Medicine, Central South University, Changsha, Hunan 410000, P. R. China

^11^FuRong Laboratory, Changsha, Hunan 410078, P. R. China

*Correspondence:

Cheng-Xian Guo, Ph D.

Center of Clinical Pharmacology, the Third Xiangya Hospital, Central South University, Changsha, 410013, Hunan, P.R. China.

Tel: +86 731 88638888

E-mail: gchxyy@163.com

Guo-Dong Liu, Ph D.

Department of Geriatric Surgery, Xiangya Hospital, Central South University, Xiangya Road 87, Changsha, Hunan, 410008, P. R. China

Tel: +86 731 89753053

E-mail: guodongliu@csu.edu.cn

Ji-Ye Yin, Ph D.

Department of Clinical Pharmacology, Xiangya Hospital, Central South University, 110 Xiangya Road, Changsha, Hunan 410078, P. R. China

Tel: +86 731 84805380

Fax: +86 731 82354476

E-mail: yinjiye@csu.edu.cn

Wei Wu, Ph D.

Department of Geriatric Surgery, Xiangya Hospital, Central South University, Xiangya Road 87, Changsha, Hunan, 410008, P. R. China

Tel: +86 731 89753053

E-mail: wuweixy@csu.edu.cn

**
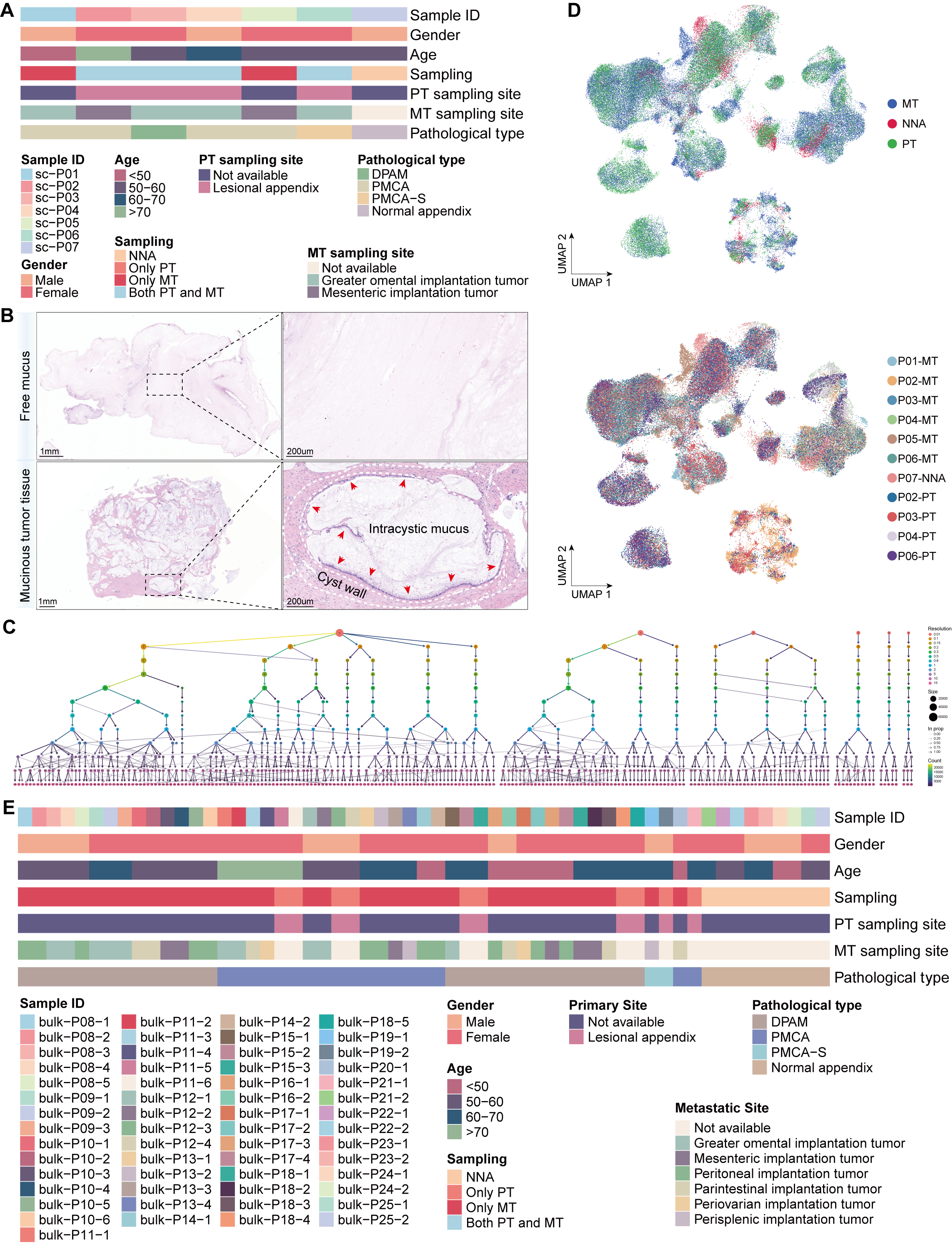
**

**Supplementary Figure 1. Clinical information of the study cohorts and overall histologic and single-cell features of PMP samples. Extension of Figure 1.** **A.** Clinical characteristics and sampling information of the 7 patients in the scRNA-seq cohort, including 6 PMP patients (P01-P06) and 1 non-PMP patient (P07). **B.** Representative HE staining images showing the histologic features of free mucus and mucinous tumor tissue in the abdominopelvic cavity. Free mucus contained almost no cellular components. In mucinous tumor tissue, tumor epithelial cells lined the inner surface of the cyst wall, while the cystic lumen was filled with intracystic mucus. Red arrows indicate the tumor epithelial cells. Scale bars, 1 mm (left) and 200 µm (right). **C.** Cluster-tree visualization of unsupervised clustering results across multiple Seurat resolution parameters. **D.** UMAP plots showing overall cell distributions from the scRNA-seq cohort across tissue origins (top) and individual samples (bottom). **E.** Clinical characteristics and sampling information of the independent bulk RNA-seq validation cohort, comprising 57 samples from 18 patients, including 14 PMP patients (P08-P21) and 4 non-PMP patients (P22-P25).


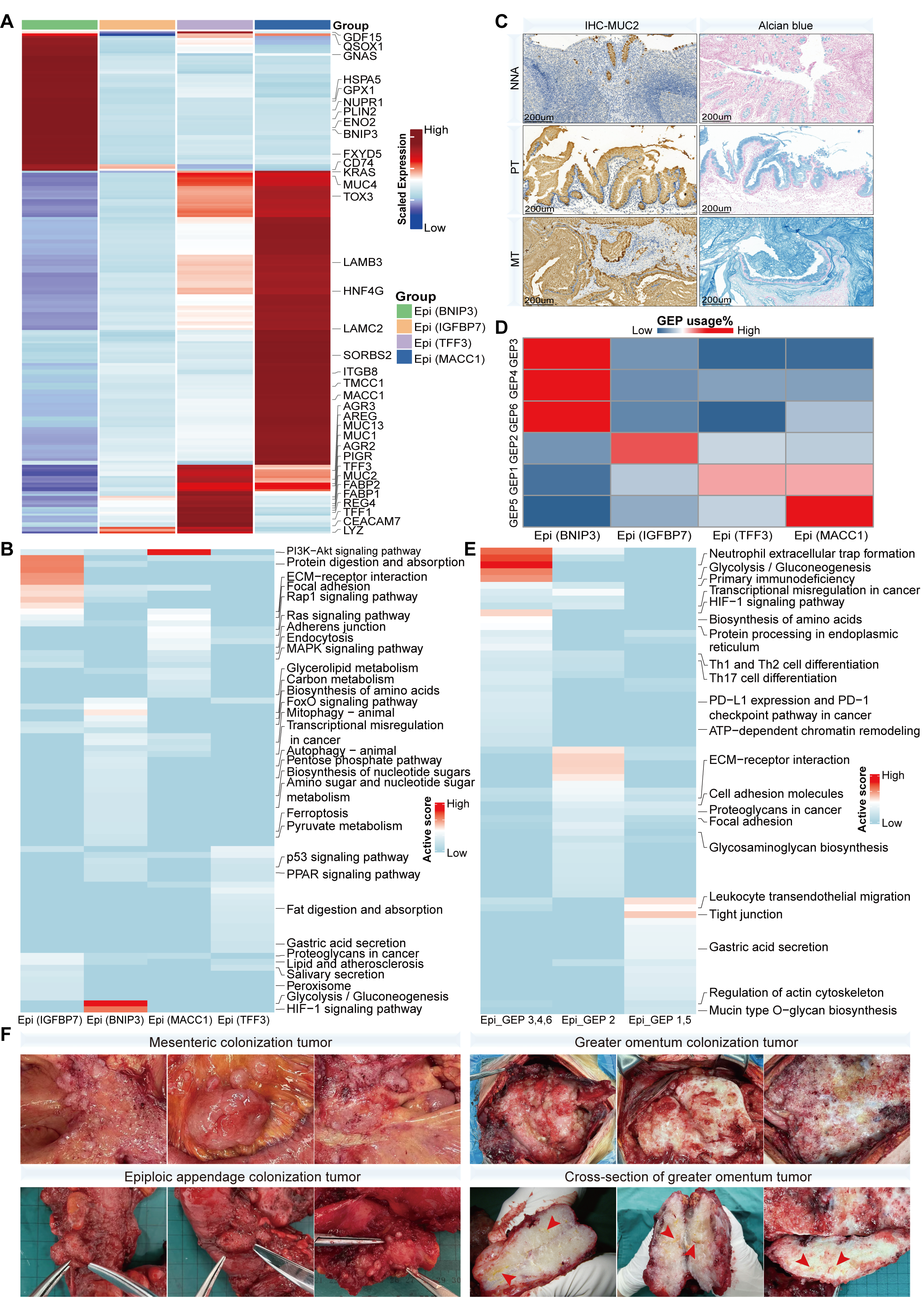


**Supplementary Figure 2. Functional annotation and metabolic features of epithelial subclusters. Expansion of Figure 2. A.** Heatmap showing the scaled expression of differentially expressed genes across epithelial cell subclusters. **B.** Heatmap showing KEGG pathway enrichment across epithelial cell subclusters. **C.** Representative images of MUC2 immunohistochemical staining (left) and Alcian blue staining (right) in NNA, PT, and MT samples. Scale bars, 200 µm. **D.** Heatmap showing the usage patterns of six GEPs identified by cNMF across epithelial cell subclusters. **E.** Heatmap showing functional pathway enrichment of distinct epithelial GEPs based on KEGG analysis. **F.** Representative intraoperative and gross images showing preferential colonization of PMP metastatic lesions at fat-rich sites, including mesentery, epiploic appendage, and greater omentum. The red arrow indicates residual fat tissue following the dissection of omental metastatic lesions.


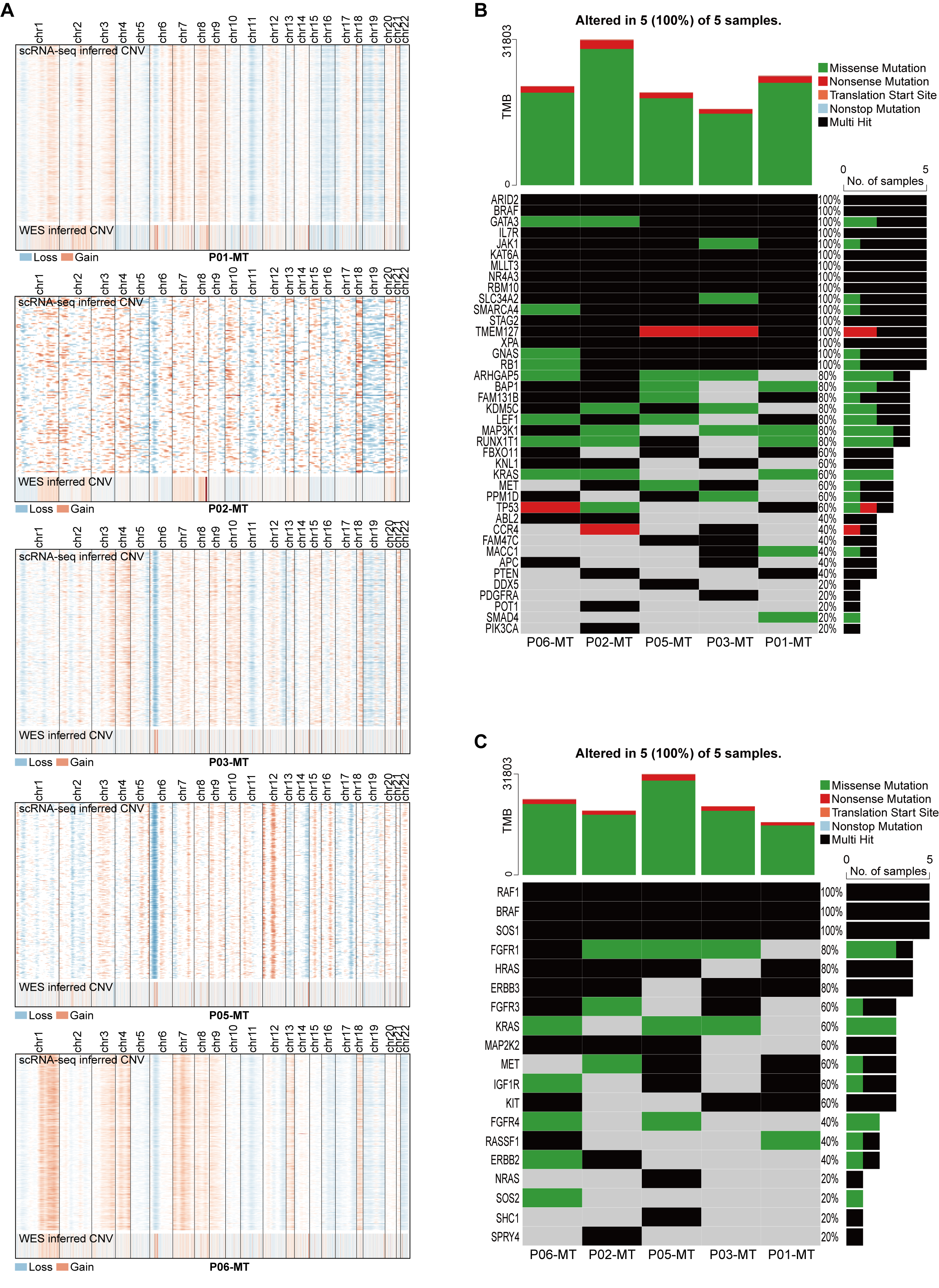


**Supplementary Figure 3. WES validation of CNV patterns and mutational landscapes in MT samples. Expansion of Figure 3.** **A.** Comparison of CNV patterns inferred from scRNA-seq (top) and WES (bottom) in MT samples from five patients (P01, P02, P03, P05, and P06). **B.** Heatmap displaying driver gene mutation profiles identified by WES in MT samples from five patients. The top bar plot indicates tumor mutational burden for each sample, and the right bar plot indicates the proportion of samples harboring alterations in each gene. **C.** Mutation status of RAS pathway-associated genes identified by WES in MT samples from five patients.


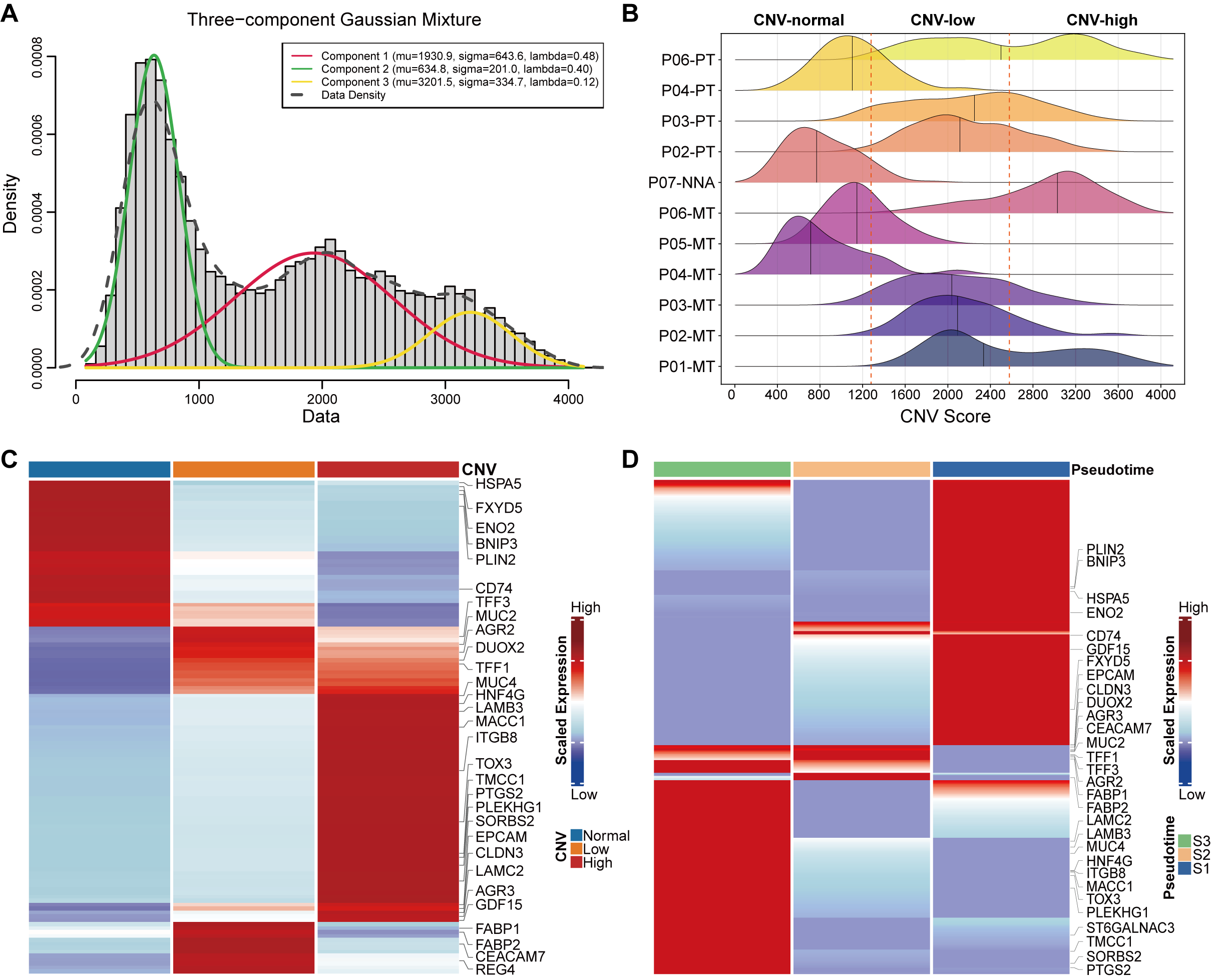


**Supplementary Figure 4. CNV stratification and pseudotime-associated transcriptional programs in epithelial cells. Expansion of Figure 3. A.** Distribution of inferCNV-derived CNV accumulation scores across all epithelial cells pooled from the scRNA-seq dataset, fitted with a three-component Gaussian mixture model. **B.** Density distributions of epithelial CNV accumulation scores for each individual sample. Dashed vertical lines indicate the two global thresholds used to define CNV-normal, CNV-low, and CNV-high groups. **C-D.** Heatmap showing scaled expression of differentially expressed genes across epithelial cells grouped by inferred CNV burden (C) and pseudotime state (D).


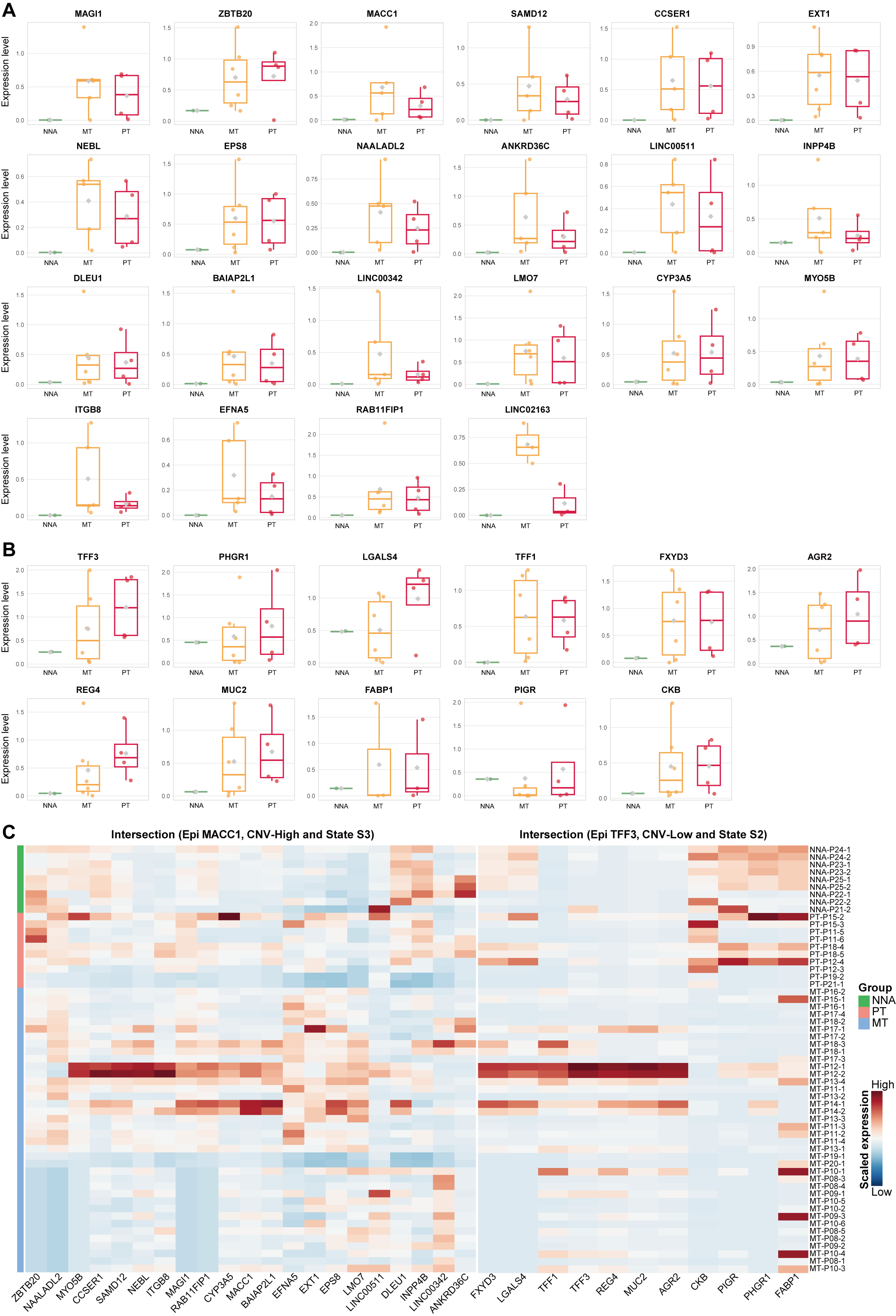


**Supplementary Figure 5. Expression patterns of malignant state- and mucus secretion-associated epithelial gene sets. Extension of Figure 3.** **A-B.** Expression levels of malignant state-associated intersecting genes identified from Epi (MACC1), CNV-high, and pseudotime-associated state S3 (A), and mucus secretion-associated intersecting genes identified from Epi (TFF3), CNV-low, and pseudotime-associated state S2 (B), in epithelial cells from NNA, PT, and MT samples in the scRNA-seq cohort. Data are presented as median with IQR. Statistical analysis of the comparison between the PT (n=4) and MT (n=6) groups was performed using the Wilcoxon rank-sum test. **C.** Heatmaps showing expression patterns of malignant state-associated intersecting genes (left) and mucus secretion-associated intersecting genes (right) across samples in the bulk RNA-seq cohort (n = 57).


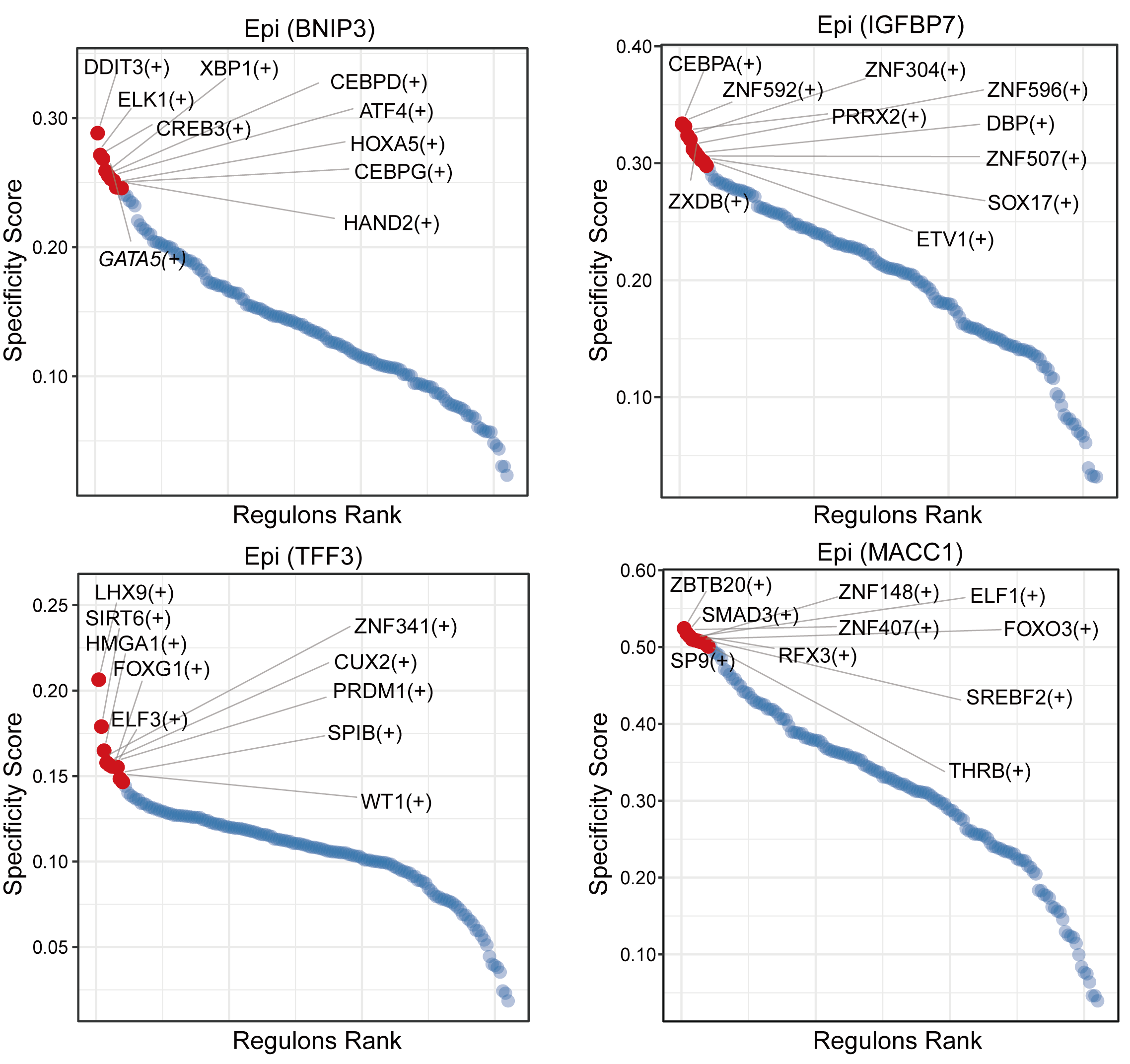


**Supplementary Figure 6. Subcluster-specific transcription factor regulons in epithelial subclusters. Extension of Figure 3.** Specificity scores and ranking of regulons in epithelial subclusters. Selected top 10-ranked subcluster-specific regulons are labeled in each panel.


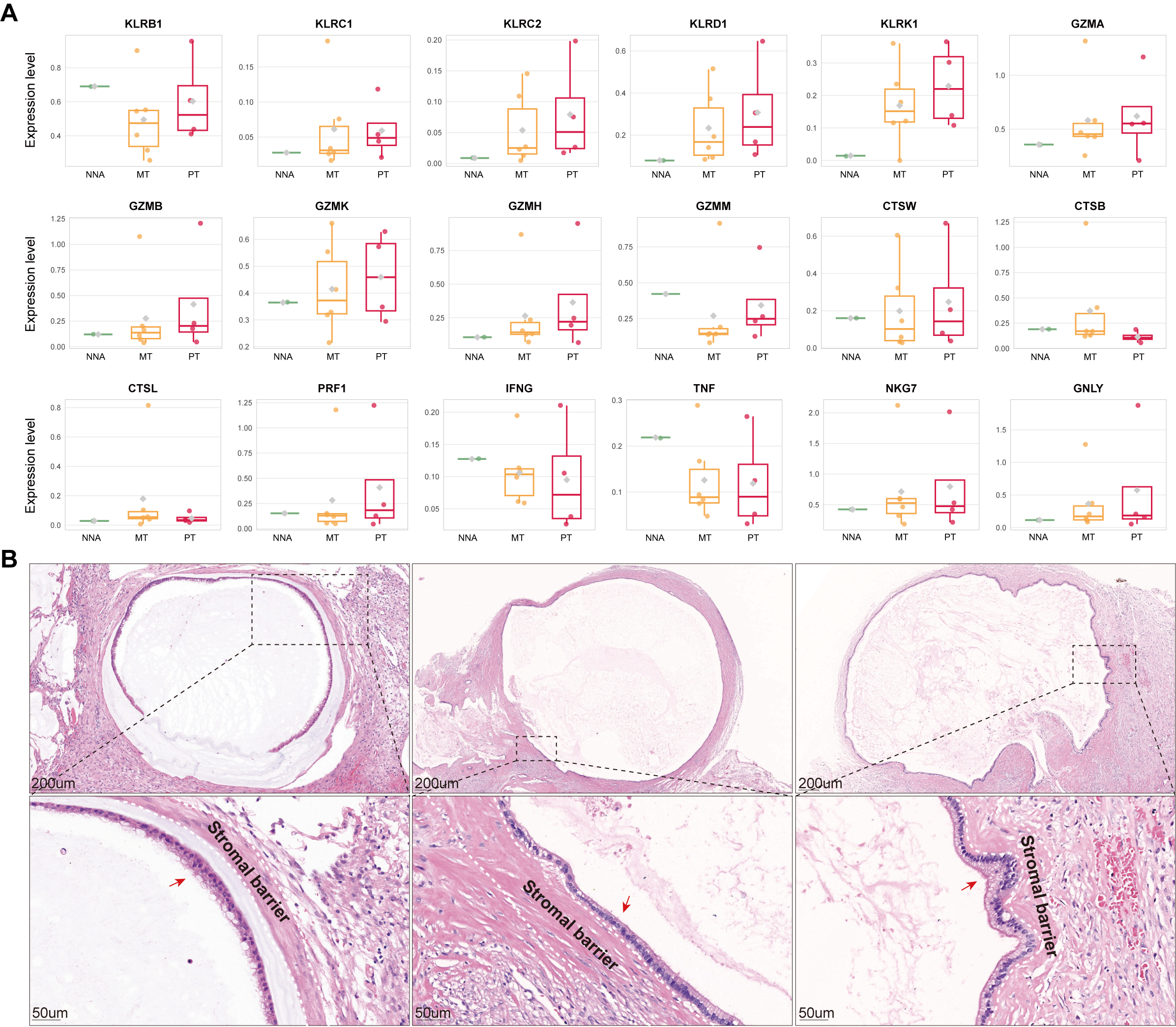


**Supplementary Figure 7. Cytotoxicity-related genes expression and histological stromal barriers. Extension of Figure 4.** **A.** Expression levels of cytotoxicity-related genes in T cells from NNA, PT, and MT samples in the scRNA-seq cohort. Data are presented as median with IQR. Statistical analysis of the comparison between the PT (n=4) and MT (n=6) groups was performed using the Wilcoxon rank-sum test. **B.** Representative HE staining of MT showing tumor epithelial cells (red arrows) lining the inner surface of cystic structures and a dense outer stromal barrier (white dashed box) surrounding the tumor cells. Scale bars, 200 µm (top) and 50 µm (bottom).


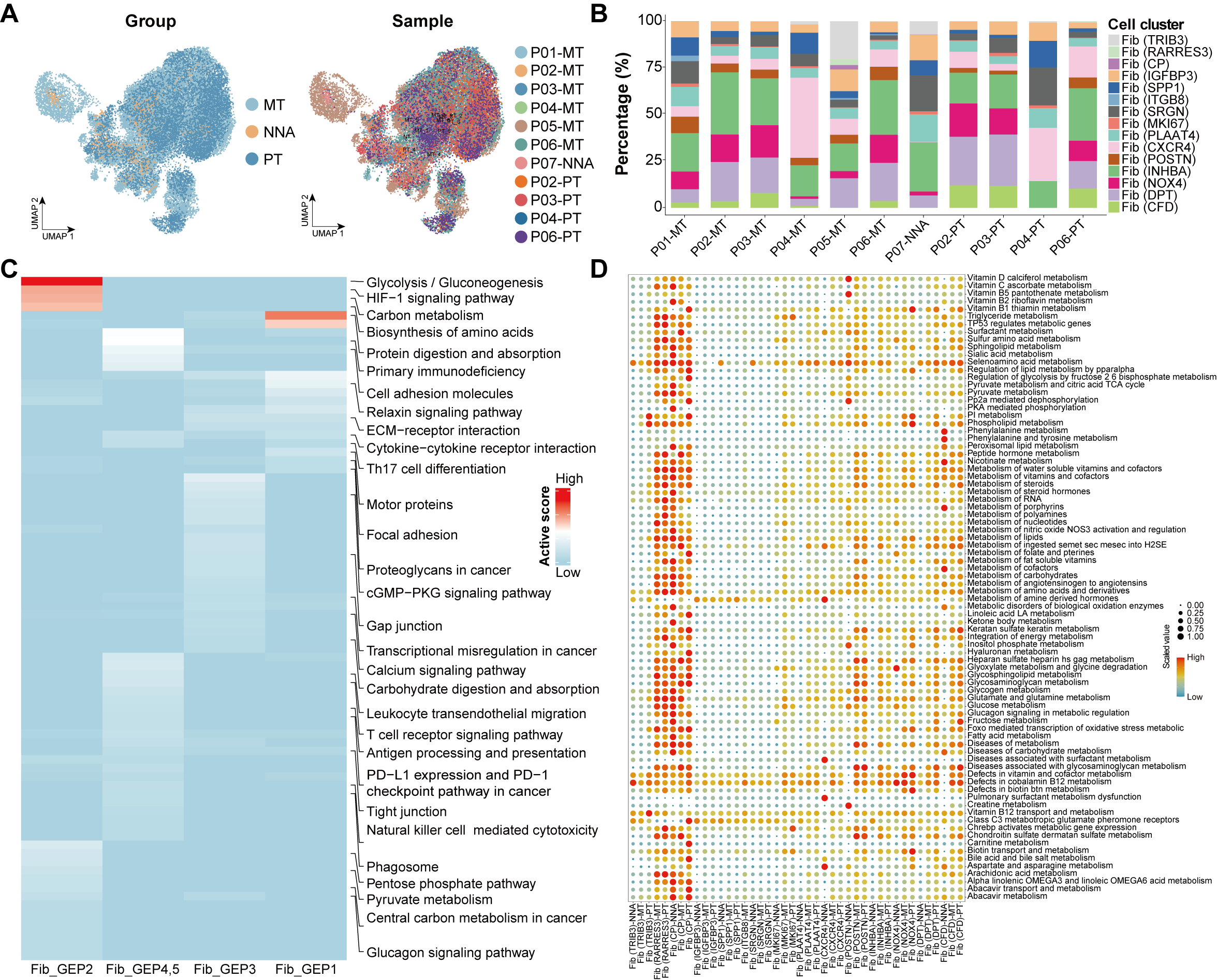


**Supplementary Figure 8. Additional characterization of fibroblast subclusters, gene expression programs, and metabolic activities. Extension of Figure 5.** **A.** UMAP plot showing fibroblast distributions across tissue origins (left) and individual samples (right). **B.** Stacked bar plot shows the relative proportion of fibroblast subclusters across individual samples. **C.** KEGG pathway enrichment analysis of distinct GEPs in fibroblasts. **D.** Dot plot illustrating metabolic pathway activity of fibroblast subclusters across tissue origins based on scMetabolism analysis.


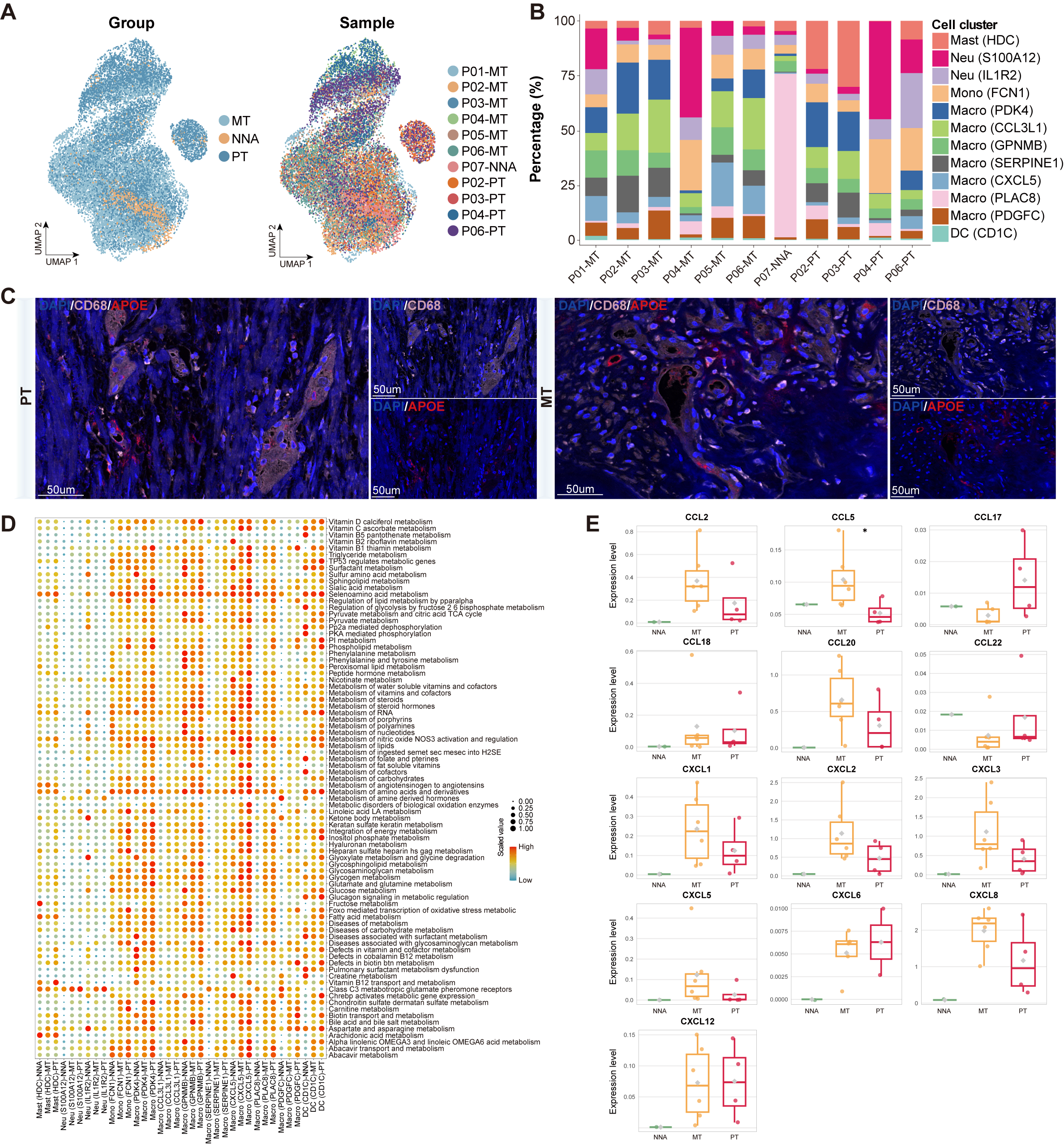


**Supplementary Figure 9. Additional characterization of myeloid subclusters, metabolic activities, and chemokine expression. Extension of Figure 6.** **A.** UMAP plots illustrating myeloid cell distributions across tissue origins and individual samples. **B.** Stacked bar plot shows the relative proportion of myeloid subclusters across individual samples. **C.** mIHC showing colocalization of DAPI (blue), CD68 (pink), and APOE (red) in PT and MT. Scale bars, 50 µm. **D.** Dot plot illustrating metabolic pathway activity of myeloid subclusters across tissue origins based on scMetabolism analysis. **E.** Expression levels of selected chemokines in myeloid cells from NNA, PT, and MT samples in the scRNA-seq cohort. Data are presented as median with IQR. Statistical comparison between the PT (n=4) and MT (n=6) groups was performed using the Wilcoxon rank-sum test. **P* < 0.05.


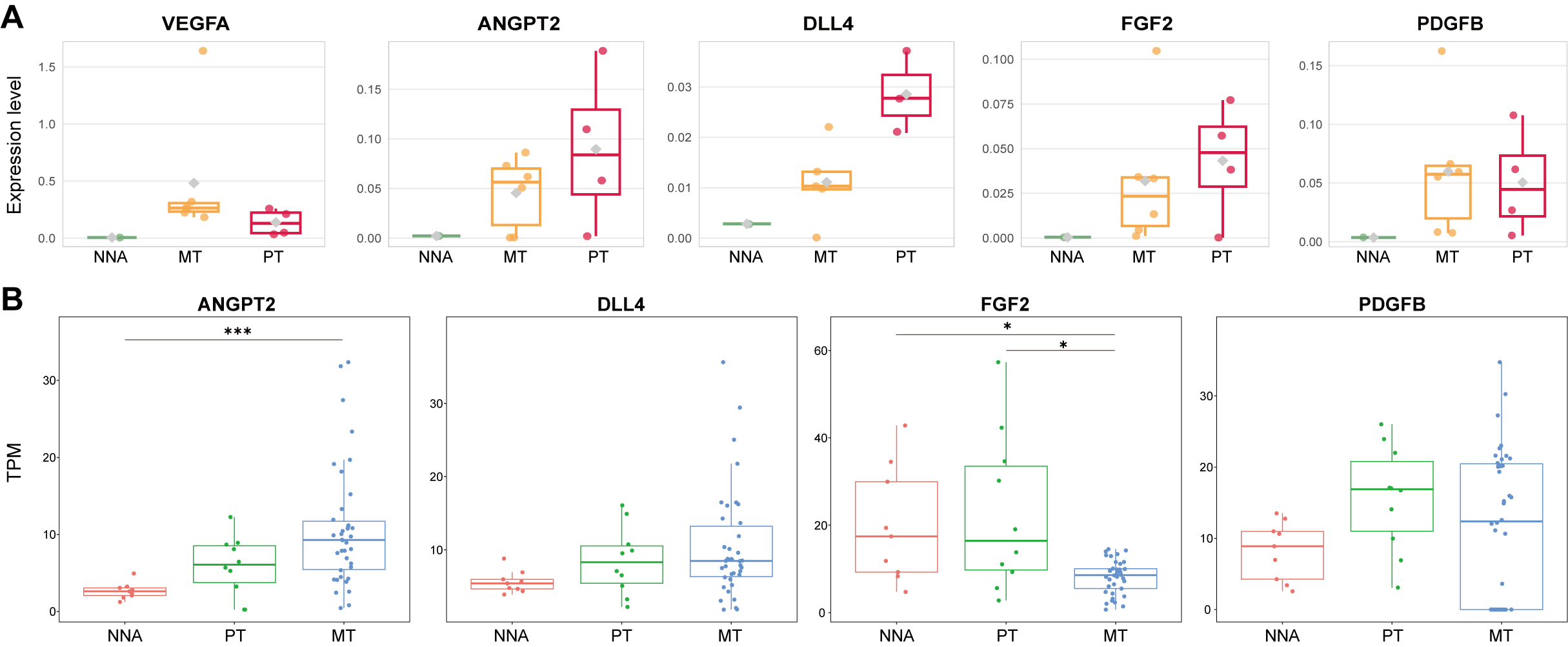


**Supplementary Figure 10. Expression of pro-angiogenic factors in scRNA-seq and bulk RNA-seq cohorts. Extension of Figure 7. A.** Expression levels of selected pro-angiogenic factors across NNA, PT, and MT samples in the scRNA-seq cohort. Data are presented as median with IQR. Statistical comparison between the PT (n=4) and MT (n=6) groups was performed using the Wilcoxon rank-sum test. **B.** Box plots showing the expression of selected pro-angiogenic factors across tissue origins in an independent bulk RNA-seq cohort (NNA = 9, PT = 10, and MT = 38). Data are presented as median with IQR. Statistical significance was assessed using the Kruskal-Wallis test followed by Dunn’s multiple-comparisons test. **P* < 0.05, ****P* < 0.001.
